# Predicting intrathecal immunoglobulin synthesis in the ICU: a comparative study of IgG-based indexes

**DOI:** 10.1186/s13613-025-01475-7

**Published:** 2025-04-30

**Authors:** Alexander Balcerac, Clémence Marois, Delphine Sterlin, Benjamin Rohaut, Sophie Demeret, Nicolas Weiss, Loic Le Guennec

**Affiliations:** 1 AP-HP.Sorbonne Université, Faculté de Médecine, Sorbonne Université, Hôpital de la Pitié-Salpêtrière, 47-83 Boulevard de l’Hôpital, 75013 Paris, France; 2 Médecine Intensive Réanimation à Orientation Neurologique, Département de Neurologie, Hôpital de la Pitié-Salpêtrière, AP-HP.Sorbonne Université, Assistance Publique-Hôpitaux de Paris, 47-83 Boulevard de l’Hôpital, 75013 Paris, France; 3 DMU Neuroscience, Institut de Neurosciences Translationnelles IHU-A-ICM, 47-83 Boulevard de l’Hôpital, 75013 Paris, France; 4 Groupe de Recherche Clinique en REanimation et Soins Intensifs du Patient en Insuffisance Respiratoire aiguE (GRC-RESPIRE) Sorbonne Université, Paris, France; 5 Inserm, Centre d’immunologie et des Maladies Infectieuses (CIMI-Paris), 83 Boulevard de l’hôpital, 75013 Paris, France; 6 Département d’immunologie, Assistance Publique Hôpitaux de Paris (AP-HP), Hôpital Pitié-Salpêtrière, 83 Boulevard de l’hôpital, 75013 Paris, France; 7 Brain Institute - ICM, Sorbonne Université, Inserm U1127, CNRS UMR 7225, 75013 Paris, France; 8 Brain Liver Pitié-Salpêtrière (BLIPS) Study Group, INSERM UMR_S 938, Centre de recherche Saint-Antoine, Maladies metaboliques, biliaires et fibro-inflammatoire du foie, Institute of Cardiometabolism and Nutrition (ICAN), Paris, France

**Keywords:** Encephalitis, Myelitis, Autoimmune diseases, Blood-Brain-Barrier, Albumin quotients, IgG index, Reiber’s formula, Oligoclonal bands, Intrathecal immunoglobulin synthesis

## Abstract

**Background:**

Central nervous system autoimmune diseases (CNS-AD) such as autoimmune encephalitis and myelitis are severe conditions, often requiring ICU admission. Early diagnosis is crucial but difficult, as initial steps facing sub-acute neurological disorders try to exclude non-immune causes such as stroke or infections through MRI and multiplex PCR assays. Current acute-phase autoimmune identifiers are lacking, with definitive diagnosis hinging on delayed tests like antibody detection or intrathecal immunoglobulin synthesis (ISI) identification via iso-electric focalization (IEF).

This study evaluates surrogate markers, such as the IgG quotient (QIgG), IgG index, and Reiber’s formula, which are rapidly obtainable, to quickly predict ISI in the ICU setting, aiming to expedite treatment initiation.

**Methods:**

We screened all neuro-ICU admissions from 2008 to 2022 in our center, including patients who underwent a lumbar puncture (LP) and were tested for ISI via IEF. We excluded those lacking concomitant CSF/serum albumin and IgG data. Patients were categorized by final diagnosis as “CNS-AD” or “other”, and whether ISI was present. We calculated QIgG, IgG index, and Reiber’s formula, comparing their performance to IEF for sensitivity (Se) and specificity (Sp).

**Results:**

ISI was detected in 35% of patients (93/266). In the “CNS-AD” group, 54% were ISI-positive, while 21% of patients in the “Other” group also showed ISI. Among the three indexes, only the IgG index showed strong specificity (95%) but moderate sensitivity (56%). QIgG and Reiber’s formula had similar sensitivity (67% and 66%) but lower specificity (41% for both). Multivariable analysis identified age < 50 years (OR 2.5 [95% CI 1.3–4.7]) and an IgG index > 0.7 (OR 14.2 [95% CI 6.6–32.0]) as factors independently associated with ISI positivity. Using the Youden index and likelihood ratio, we recalibrated thresholds to improve performance. A “grey zone” was defined for the IgG index (0.67–0.80), below which ISI was unlikely and above which it was considered probable.

**Conclusion:**

While the IgG index’s low sensitivity limits its standalone diagnostic use, its high specificity makes this index a good one when positive, to weigh in the decision-making process to treat or not a patient with suspected CNS-AD, while awaiting IEF results, which can take days or even weeks in some centers.

**Supplementary Information:**

The online version contains supplementary material available at 10.1186/s13613-025-01475-7.

## Introduction

Central nervous system (CNS) autoimmune diseases (AD) are severe, life-threatening neurological disorders often requiring intensive care unit (ICU) admission [1]. CNS-AD covers a broad spectrum of diseases with heterogenous clinical presentation including autoimmune encephalitis (AE), neuromyelitis optica (NMO), CNS vasculitis, acute disseminated encephalomyelitis (ADEM), or myelitis. These conditions are associated with significant morbidity and mortality [1]. Thus, rapid recognition, accurate triage, and timely empiric immunomodulatory therapy of ICU patients with suspected CNS-AD are paramount [2].

When a patient presents with an acute neurological syndrome, MRI is typically the first-line diagnostic tool to rapidly rule out stroke, or spinal cord compression in cases of suggestive symptoms. If MRI findings exclude these diagnoses, clinicians must then consider the possibility of encephalitis or myelitis and face the challenge of differentiating between infectious and autoimmune etiologies. For infectious causes, lumbar puncture (LP) combined with multiplex PCR assays can quickly identify many common pathogens, providing rapid diagnostic clarity [3]. However, no comparable rapid tests exist for autoimmune conditions, where confirmation relies on the detection of specific antibodies. This process can take several weeks, and in some cases, results remain inconclusive due to seronegative autoimmune encephalitis or myelitis of unknown etiology. Even the detection of oligoclonal bands (OCB) in the cerebrospinal fluid (CSF), considered as a hallmark of intrathecal synthesis of immunoglobulins (ISI) [4], and classically associated with CNS-AD, is delayed by the time-consuming process of iso-electric focalization (IEF). The lack of rapid and reliable markers to identify autoimmune etiologies in the acute phase significantly delays the initiation of appropriate immunomodulatory therapy, a delay associated with increased morbidity and mortality [5–7].

Because ISI is frequently associated with CNS autoimmune diseases, its early detection may serve as a useful indicator to guide clinical suspicion and initiate immunomodulatory treatment while awaiting more definitive results [2, 8].

Yet, surrogate methods for ISI prediction exist, offering a faster alternative to the lengthy process of IEF. These include the IgG quotient (QIgG), IgG index [9, 10], and Reiber’s formula [11], which use albumin and IgG concentrations in serum and CSF to infer ISI. These indices, obtained through nephelometry, provide results more rapidly than IEF.

However, these indirect methods were predominantly validated outside the ICU setting, particularly in patients with multiple sclerosis [12]. ICU patients exhibit distinct physiological conditions, such as systemic inflammation [13] and a high prevalence of hypoalbuminemia [14, 15], which may affect the performance of these indices.

To address this gap, our study aims to evaluate the diagnostic performance of QIgG, IgG index, and Reiber’s formula in predicting ISI in this population. In addition, as a secondary objective, we assess the ability of these indexes to discriminate CNS-AD from other diagnoses. By assessing the utility of these indexes in this specific population, we aim to improve the diagnostic process for CNS-AD and support earlier therapeutic interventions.

## Methods

### Study design and setting

We analyzed all hospital stays in neuro-ICU at l’Hôpital de la Pitié-Salpêtrière in Paris, France between January 2008 and October 2021 within our database.

Inclusion criteria were as follows: age > 18 years, and patients who underwent LP with CSF and serum IEF analysis during the study period. Exclusion criteria were as follows: absence of concomitant available biological data for albumin and IgG in patient’s CSF and serum. For each patient, the first accessible concomitant blood sample and LP data available in our registry were used.

All patients were screened for their final diagnosis at ICU discharge. Data were retrospectively collected among the patients’ files. Collected items included demographic data, comorbidities, date of disease onset, date of concomitant serum and LP accessible, CSF data, final diagnosis retained at ICU discharge or death, SAPS II (Simplified Acute Physiology Score) at ICU admission, ICU organ support, date of ICU admission and hospital discharge, and death.

For statistical analysis, patients were categorized according to their final diagnosis as “CNS-AD” and “other diagnosis”. The term CNS-AD encompassed the following diagnoses: Antibody-mediated AE, CNS demyelinating inflammatory diseases, Behçet with CNS involvement, Sarcoidosis with CNS involvement, cerebral mastocytosis, Lupus with CNS involvement and central nervous system vasculitis. In addition, for cases of encephalitis of unknown etiology, we retrospectively applied the diagnostic criteria proposed by Graus et al. (2016) [16] for autoimmune encephalitis. Patients fulfilling criteria for *definite* or *probable* autoimmune encephalitis were reclassified into the CNS-AD group, while those not meeting these criteria were retained in the “Other diagnosis” group.

Similarly, for patients with myelitis, we applied the 2002 Transverse Myelitis Consortium Working Group (TMCWG) criteria [17]. Idiopathic transverse myelitis cases fulfilling TMCWG diagnostic criteria were reclassified as CNS-AD, and other forms of myelitis remained classified as “Other”.

Patients were also categorized regarding ISI presence as “ISI-positive patients” and “ISI-negative patients”.

No sample calculation was performed, and sample size was determined by the availability of the CSF data. Patients were managed in accordance with practices recommended at the time of the study.

### Sample preparation and CSF isoelectric focalization

For the serum obtention process, patient samples were collected in tubes with clot activator and centrifuged at 2500 g for 10 min to separate the serum prior to analysis. CSF obtention was performed using simple polystyrene tubes.

Both CSF and blood albumin and IgG measurements were performed by nephelometry using the BN^TM^II.

System analyzer (Siemens Healthcare). The identification of IgG oligoclonal bands was done by IEF on agarose gel using the Hydragel CSF isofocusing kit (Sebia^®^). Both CSF and blood samples were prepared simultaneously and applied to a sample application alongside a control from a patient with positive oligoclonal bands. The presence of oligoclonal bands was defined as two or more clear immunoglobulin bands present in the CSF but absent from the blood sample.

### Cerebro-spinal fluid index scores

The three main index scores for ISI prediction from the literature were used: the IgG quotient (QIgG) [18], IgG index [9] and Reiber’s formula [11]. These scores were calculated using the following formulas:

1/QIgG:

$$QIgG = \frac{CSF\,IgG}{{Serum\,IgG}}$$

According to the literature, QIgG was considered abnormal and predictive of ISI if > 0.0035 [19].

2/IgG Index:

$$IgG\,Index = \left\{ {\frac{{\frac{CSF\,IgG}{{Serum\,IgG}}}}{{ \frac{CSF\,Albumin}{{Serum\,Albumin}}}}} \right\}$$

According to the literature, the IgG index was considered abnormal and predictive of ISI if > 0.7 [20].

3/Reiber’s hyperbolic formula:

$$Reiber^{\prime}s\,formula = Serum\,Ig*\left\{ {QIgG\,-\,Qlim} \right\}$$

$$with\,{ }Q_{lim} = { }0,93{ }\sqrt {\left\{ {\left( {Q_{Alb} } \right)^{2} \cdot 6{ } \cdot 10^{{\left\{ { - 6} \right\}}} } \right\}} - { }1,7{ } \cdot 10^{3}$$

$$and\,QAlb = \frac{{4{ } + \frac{{\text{ age }}}{{{ }15}}}}{1000}$$

Reiber's formula evaluates intrathecal synthesis by comparing QIgG with a calculated threshold, QLim. Qlim, or “QIgG upper limit” is a hyperbolic threshold that accounts for the albumin quotient (QAlb). A QIgG ≤ QLim suggests no intrathecal synthesis (normal values), while a QIgG > QLim indicates probable ISI, as the IgG concentration in the CSF exceeds the expected diffusion from the serum [11].

### Data analysis

All statistical analyses were performed using GraphPad Prism (version 8.4.2; GraphPad Software). Continuous variables were compared with Student’s t-test or the Wilcoxon signed rank test, as appropriate. Categorical variables were compared using the chi-square test for equal proportions. A *p-*value below 0.05 was chosen for statistical significance. Quantitative variables were expressed as medians with interquartile ranges [25–75 th percentile]. The diagnostic performance of each index was assessed using sensitivity (Se), Specificity (Sp), Positive Predictive Value (PPV) and Negative Predictive Value (NPV). In addition, we computed the Youden Index (defined as Se + Sp − 1) for each of the three markers—QIgG, IgG index, and Reiber’s formula—in order to determine the optimal cut-off corresponding to the maximum Youden Index value. We also computed likelihood ratios (LR) for indexes thresholds. Based on these values, we defined a diagnostic “grey zone” to improve clinical interpretation: the lower bound was determined using the optimal cut-off derived from the Youden Index, while the upper bound corresponded to the first threshold with a positive likelihood ratio (LR +) greater than 10. Results below the grey zone were interpreted as “unlikely”, those within the grey zone as “intermediate”, and those above the grey zone as “likely” for intrathecal synthesis.

The *p-value* corresponds to the two-tailed probability that the observed AUC (Area Under Curve) could occur under the null hypothesis (true AUC = 0.5). A threshold of *p* < *0.05* using a binomial exact model was considered significant to conclude that the test distinguishes patients with and without oligoclonal bands [21, 22]. ROC curves were plotted for all three indexes (IgG index, Reiber’s formula and QIgG), using IEF results (primary objective, with ISI positive = 1, and ISI negative = 0) and final diagnosis (secondary objective, with CNS-AD = 1, and other diagnosis = 0).

We also assessed whether the timing of immunosuppressive treatment (before or after the ISI workup) affected IEF results. Patients were stratified according to treatment timing, and comparisons of ISI positivity rates were performed using chi-square tests within each diagnostic group (CNS-AD vs. Other).

To assess if these indexes were associated with ISI by IEF or CNS-AD, a univariable logistic-regression model was used. Thereafter, multivariable logistic-regression models with backward-stepwise variable elimination (variable exit threshold set at p > 0.05) compared the factors that were significant in the univariable analyses. For univariable and multivariable analyses, continuous variables were dichotomized according to their median values in the studied population.

## Results

### General characteristics of patients

During the study period, 840 patients admitted to ICU underwent a LP, of whom 403 had ISI screening by IEF, and 266 patients had full data available for both CSF/serum albumin and IgG to calculate indexes (Supplementary Fig. 1 shows the study flowchart). We reported the final diagnosis of all 840 patients who underwent a LP and the ISI frequency according to the final diagnosis of the 403 patients in Table 1. Among these 403 patients, assessed only with IEF, OCBs were found in 54% of the “CNS-AD” group and 46% of patients with “CNS-AD” did not present OCBs in the CSF. For the “other diagnosis” group, OCBs were found in 21% of patients.

**Table 1:** Final diagnosis of the 840 neuro-ICU patients who underwent a lumbar puncture, and ISI screening results for the 403 patients

Final diagnosis	Patients with LP* n* = *840*	Patients with ISI screening *n* = *403*	ISI positive patients *n* = *132/403 (33%)*
**CNS-AD**	**n = 234 (28%)**	**n = 141 (35%)**	**n = 76/141 screened (54%)**
*Antibody-mediated acute encephalitis*			
Anti-NMDAr	42	29	22 (76)
Anti-GAD65	6	3	3 (100)
Anti-LGI1	3	2	0 (0)
Anti-CASPR-2	2	2	0 (0)
Anti-VGKC*	3	N/D	N/D
Anti-IgLON5	2	2	0 (0)
Anti-GFAP	2	2	2 (100)
Anti-GABA-A	1	1	0 (0)
Anti-GABA-B	1	N/D	N/D
Anti-DR2	1	1	1 (100)
*Seronegative CNS-AD*			
Definite autoimmune encephalitis	10	5	1 (20)
Possible autoimmune encephalitis	44	35	14 (40)
Idiopathic acute transverse myelitis	15	8	4 (50)
* CNS Demyelinating inflammatory diseases*			
Multiple sclerosis	37	10	9 (90)
ADEM	15	8	5 (62)
Neuromyelitis optica	19	11	5 (45)
MOG antibody-associated encephalomyelitis	4	4	1 (25)
*Systemic AD with CNS involvement*			
Behçet’s disease	3	3	2 (66)
Sarcoidosis	5	4	3 (75)
Cerebral mastocytosis	1	1	1 (100)
Cerebral histiocytosis	1	1	1 (100)
Lupus	6	3	1 (33)
Sarcoidosis	1	N/D	N/D
Central nervous system vasculitis	9	6	1 (16)
*Other CNS-AD*			
Rasmussen encephalitis	1	N/D	N/D
**Other diagnosis**	**n = 606 (72%)**	**n = 262 (65%)**	**n = 56/262 screened (21%)**
* CNS affection of unknown etiology*			
Encephalitis of unknown etiology	59	30	9 (30)
Myelitis of unknown etiology	14	7	4 (57)
Aseptic meningitis	22	16	7 (43)
* Cerebrovascular diseases*			
PRES	13	7	0 (0)
Ischemic stroke and anoxic encephalopathy	33	8	0 (0)
Brain hemorrhage	8	1	0 (0)
Arteriovenous malformation	1	1	0 (0)
*Viral infections*			
HSV-1 encephalitis	14	2	0 (0)
VZV encephalitis	9	4	3 (75)
HSV-2 encephalitis	3	2	2 (100)
HHV-6 encephalitis	4	2	0 (0)
EBV myelitis	1	N/D	N/D
HIV encephalitis	7	3	3 (100)
JCV encephalitis**	4	2	2 (100)
Dengue encephalitis	1	N/D	N/D
COVID-19 associated encephalitis	15	10	0 (0)
*Bacterial infections*			
Brain abscess/brain emboli	3	1	0 (0)
Q Fever meningoencephalitis	1	1	0 (0)
Bacterial meningitis	23	N/D	N/D
Tuberculous meningoencephalitis	21	12	5 (41)
*Fungal/Parasite infections*			
Fusobacterium spp	1	N/D	N/D
Aspergillus spp	3	2	0 (0)
Neurocryptococcosis	1	N/D	N/D
Neurotoxocariasis	5	3	1 (33)
Neurocysticercosis	1	N/D	N/D
Neurotoxoplasmosis	2	N/D	N/D
Cerebral malaria	1	1	1 (100)
* Tumoral involvement of nervous system*			
Glioma	17	9	0 (0)
Primary CNS lymphoma	9	7	4 (57)
B-Cell systemic lymphoma	8	7	3 (42)
T-Cell systemic lymphoma	2	2	2 (100)
Hodgkin lymphoma	1	1	0 (0)
Chronic lymphocytic leukemia	2	1	0 (0)
Acute myeloid leukemia	1	1	0 (0)
Myelofibrosis	1	N/D	N/D
Bing-Neel syndrome	1	1	1 (100)
Graft versus host	1	1	0 (0)
Brain metastasis of solid cancer	12	4	0 (0)
*Inflammatory polyradiculopathies*			
Lyme polyradiculitis	2	N/D	N/D
Subacute polyradiculitis	15	3	0 (0)
Guillain–Barre syndrome	64	33	1 (3)
CANOMAD	1	N/D	N/D
CIDP	17	9	0 (0)
Sarcoidosis polyradiculopathy	2	1	0 (0)
Parsonage-Turner syndrome	1	1	1 (100)
*Miscellaneous*			
Neurodegenerative disease	30	15	0 (0)
Functional neurological disorders	26	12	0 (0)
Creutzfeldt-Jakob	8	5	1 (20)
Toxic Metabolic disorders + IEM	50	19	3 (16)
Seizure (non-immune related)	58	13	4 (30)
Myasthenia gravis	5	1	0 (0)
Botulism	1	N/D	N/D
Lambert-Eaton	1	1	1 (100)

Results are expressed as number (%). *n* number of patients, *ICU* Intensive Care Unit, *CNS* Central Nervous System, *AD* autoimmune disease, *ISI* Intrathecal Synthesis of Immunoglobulins, *IEM* Inborn error of metabolism, *ADEM* Acute disseminated encephalomyelitis, *MOG* Myelin oligodendrocyte glycoprotein, *CANOMAD* chronic ataxic neuropathy, ophthalmoplegia, immunoglobulin M paraprotein, cold agglutinins, and disialosyl antibodies, *CIDP* Chronic inflammatory demyelinating polyneuropathy, *NMDAr* N-methyl-D-aspartate receptor, *GAD65* glutamic acid decarboxylase 65, *LGI1* Leucine-rich glioma-inactivated protein 1, *CASPR-2* Contactin-associated protein-like 2, *VGKC* voltage-gated potassium channel, *IgLON5* IgLON Family Member 5, *GFAP* Glial fibrillary acidic protein, *GABA* γ-Aminobutyric acid, *DR2* Dopamine Receptor 2, *AI* autoimmune, *HHV-6* Human herpesvirus 6, *HSV* Herpes simplex virus, *VZV* Varicella zoster virus, *EBV* Epstein–Barr virus, *JCV* John Cunningham virus, *HIV* human immunodeficiency virus, *COVID-19* coronavirus disease 2019, *N/D* No data, *PRES* Posterior reversible encephalopathy

^*^Anti-VGKC antibodies were identified before the discovery of LGi1 and CASPR2 antibodies

^**^Among the four patients with JCV encephalitis: 3 had HIV, and 1 was treated with Fingolimod for MS

Baseline characteristics of the 266 patients with full data available are reported in Table 2. Among these 266 patients, the median age was 50 years [IQR: 32–63] with 42% being women (n = 133/266 patients). Immunodepression was found in 32 patients (12%), including 2 cases of solid cancers (1%), 16 hematologic malignancies (6%), 5 transplant recipients (2%), and 11 patients with human immunodeficiency virus (HIV) (4%). The median SAPS II at admission was 26 [IQR: 13–44] and 41% of patients had invasive mechanical ventilation (MV). Among ventilated patients, the median length of MV was 32 days [IQR: 12–53]. Median length of stay (LOS) in ICU was 15 days [IQR: 6–37] with an overall ICU-mortality of 9%. The median time between disease onset and first available concomitant blood/CSF Albumin and IgG result was 20 days [IQR; 7–73]. Lumbar puncture found median leukocytes count at 4/mm3 [IQR: 1–17], erythrocytes at 6/mm3 [IQR: 1–75], protein level at 0.5 g/l [IQR: 0–1], glucose level at 3.7 mmol/l [IQR: 3–4], albumin level at 260 mg/l [IQR: 165–447] and IgG level at 50 mg/l [IQR: 29–91]. Blood sample found a median albumin level at 36 g/l [IQR: 28–43], and IgG level at 11 g/l [IQR: 8–15].

**Table 2:** Characteristics of the 266 patients with complete data and comparison of CSF index scores between ISI-positive and ISI-negative patients

Characteristics	Full population n = 266	ISI-positive patients n (%) = 93 (35)	ISI-negative patients n (%) = 173 (65)	p-value
Age, years	50 [32–63]	38 [28–54]	54 [38–67]	** < 0.001**
Sex, female	113 (42)	45 (48)	68 (39)	0.153
Immunodepression	32 (12)	15 (16)	17 (10)	0.132
Solid cancer	2 (1)	1 (1)	1 (0,5)	0.654
Hemopathy	16 (6)	3 (3)	13 (8)	0.161
Transplantation	5 (2)	3 (3)	2 (1)	0.236
HIV	11 (4)	10 (11)	1 (0,5)	** < 0.001**
SAPS2 II at ICU admission	26 [13–44]	23 [13–38]	26 [13–45]	0.100
Immunosuppressive treatment administered*	156 (59%)	63 (68%)	93 (53%)	**0.027**
Mechanical ventilation (invasive)	110 (41)	34 (37)	76 (44)	0.244
Length of mechanical ventilation (days)	32 [12–53]	36 [12–65]	31 [14–52]	0.369
Length of stay in ICU (days)	15 [6–37]	19 [8–45]	14 [6–35]	0.091
Death in ICU	24 (9)	6 (6)	18 (10)	0.283
Time between onset and ISI workup (days)	20 [7–73]	32 [11–101]	17 [6–67]	0.053
LP findings during ISI workup**				
Leukocytes (/mm3))	4 [1–17]	7 [2–35]	2 [0–9]	** < 0.001**
Erythrocytes (/mm3)	6 [1–75]	3 [1–38]	7 [1–98]	0.095
Protein (g/l)	0,5 [0–1]	0,5 [0–1]	0,5 [0–1]	0.699
Glucose (mmol/l)	3,7 [3, 4]	3,8 [3–5]	3,7 [3, 4]	0.175
Albumin level (mg/l)	260 [165–447]	282 [189–479]	250 [152–395]	0.051
IgG level (mg/l)	50 [29–91]	58 [36–137]	46 [26–80]	**0.008**
Blood sample during ISI workup				
Albumin level (g/l)	36 [28–43]	35 [28–42]	38 [29–44]	0.157
IgG level (g/l)	11 [8–15]	11 [8–16]	11 [8–14]	0.492
CSF index scores				
QIgG > 0.0035	163 (62)	62 (67)	101 (59)	0.218
IgG index > 0.7	64 (24)	53 (57)	11 (6)	** < 0.001**
Reiber’s formula > 0 ***	150 (56)	59 (63)	91 (34)	0.089

Results are expressed as n (%) or median [25 th;75 th percentile]. *ICU* Intensive Care Unit, *ISI* intrathecal Synthesis of Immunoglobulins, *CSF* Cerebro Spinal Fluid, *CNS* Central Nervous System, *AD* Autoimmune Disease, *HIV* Human Immunodeficient Virus, *LP* Lumbar puncture, *SAPS* Simplified Acute Physiology Score, *IgG* Immunoglobulin G, *QIgG* Quotient IgG, *QLim* QIgG upper Limit

^*^Here, the association between ISI results and the decision to initiate immunosuppressive therapy is not investigated

^**^Date of first concomitant serum and LP Albumin/IgG result accessible, many patients had a prior standard LP

^***^Rebeir’s formula > 0 corresponds to QIgG > QLim

### Comparison of ISI positive patients with ISI negative patients

To evaluate the diagnostic efficacy of QIgG, IgG index, and Reiber's formula in predicting ISI in neuro-critically ill patient admitted to the ICU, patients were split into two groups (Table 2); 93 ISI positive patients (35%) and 174 ISI negative patients (65%). The ISI-positive group differed from ISI-negative group in terms of median age, which was respectively at 38 years [IQR: 28–54] and 54 years [IQR: 38–67] (p < 0.001), as well as in the prevalence of HIV, which was higher in the ISI-positive group compared to the ISI-negative group, respectively at 11% (n = 10/93 patients) and 0.5% (n = 1/173 patients) (p < 0.001). Additionally, the median leukocytes count was higher in the ISI-positive group compared to the ISI-negative group, respectively at 7/mm3 [IQR: 2–35] and 2 [IQR: 0–9] (p < 0.001).

Among the three CSF index scores used in the study, only the IgG index showed a statistically significant difference between ISI-positive group and ISI-negative groups. The IgG index was positive in 57% of ISI-positive patients (n = 53/93) and 6% of ISI-negative patients (n = 11/173) (p < 0.001). The detailed distribution of these scores for each final diagnosis is described in Supplementary Table 1.

Among the 266 patients with complete data, 156 received an immunosuppressive treatment at some point during their hospital stay (Supplementary Table 2). Among them, 117 (75%) received treatment after the ISI workup, and 39 (25%) received treatment before. In the “CNS-AD” group, the proportion of ISI-positive patients was similar between those treated before and after the ISI workup (54% vs. 52%, p = 0.851). In the “Other diagnoses” group, the proportion of ISI positivity also did not differ significantly between the two subgroups (9% vs. 24%, p = 0.263). No significant differences were found between the two groups in terms of CSF or serum parameters, including IgG index (p = 0.841), Reiber’s formula positivity (p = 0.384), or QIgG (p = 0.552). These findings suggest that the timing of immunosuppressive treatment relative to the ISI workup did not significantly influence the likelihood of detecting intrathecal immunoglobulin synthesis.

**Fig. 1:**
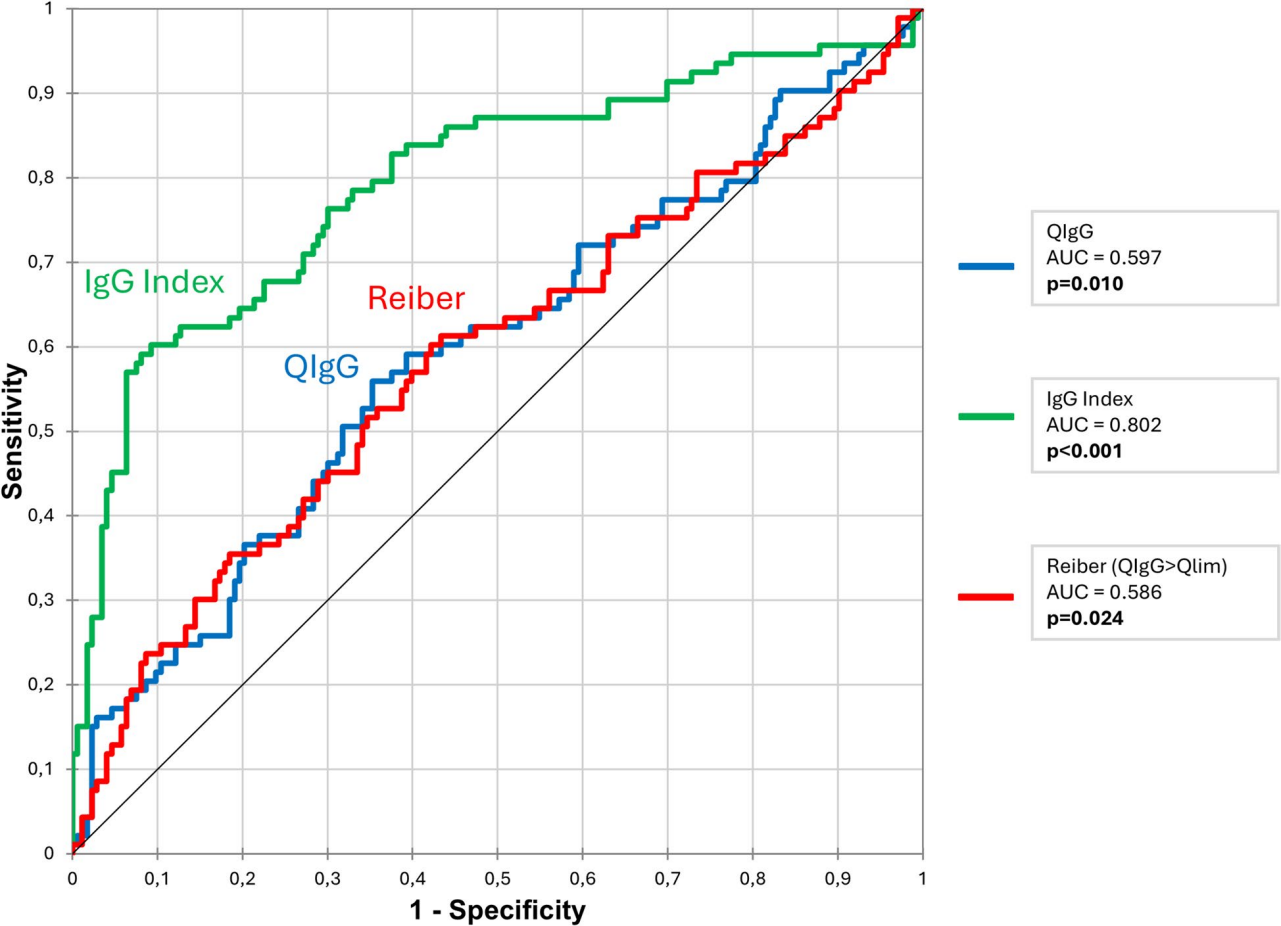
ROC curves for the prediction of ISI using the 3 indexes

The sensitivity (Se) and specificity (Sp) for each index were calculated (Supplementary Table 3); QIgG: Se 67% [95% CI 57–75], Sp 41% [95% CI 34–49]; IgG index: Se 56% [95% CI 46–66], Sp 95% [95% CI 91–98]; and Reiber’s formula: Se 66% [95% CI 57–75], Sp 41% [95% CI 34–49]. Positive Predictive Value (PPV) and Negative Predictive Value (NPV) were also assessed for these three indexes; QIgG: PPV 38% [95% CI 30–45], NPV 70% [95% CI 61–79]; IgG index: PPV 87% [95% CI 78–95], NPV 80% [95% CI 75–86]; and Reiber’s formula: PPV 37% [95% CI 30–45], NPV 70% [95% CI: 61–79]. The relationship between sensitivity and 1-specificity is illustrated in the ROC curves shown in Fig. 1.

In order to refine the diagnostic thresholds for ISI prediction, we calculated the Youden Index for each of the three CSF indexes. This analysis, illustrated in Supplementary Fig. 2, identified new optimal cut-off values: QIgG > 0.005, IgG index > 0.67, and Reiber’s formula > QLim + 5.46. Using these thresholds, we reanalyzed the population for differences between ISI-positive and ISI-negative patients (Supplementary Table 4). The revised thresholds significantly improved discriminatory performance for each index. A QIgG > 0.005 (p = 0.003), an IgG index > 0.67 (p < 0.001), and a Reiber’s formula > QLim + 5.46 (p = 0.007) were each significantly associated with ISI positivity.

### Refinement of indexes thresholds for ISI prediction

Based on the Youden Index, we defined the lower bound of a diagnostic grey zone for each index, below which ISI was considered unlikely. To determine the upper bound, we identified the first threshold associated with a positive likelihood ratio (LR +) > 10. As shown in Supplemental Fig. 2, only the IgG index met this criterion, with an upper threshold at 0.80. This defines a grey zone between 0.67 and 0.80 for ISI prediction using the IgG index; values above 0.80 were considered indicative of a probable intrathecal synthesis.

**Fig. 2:**
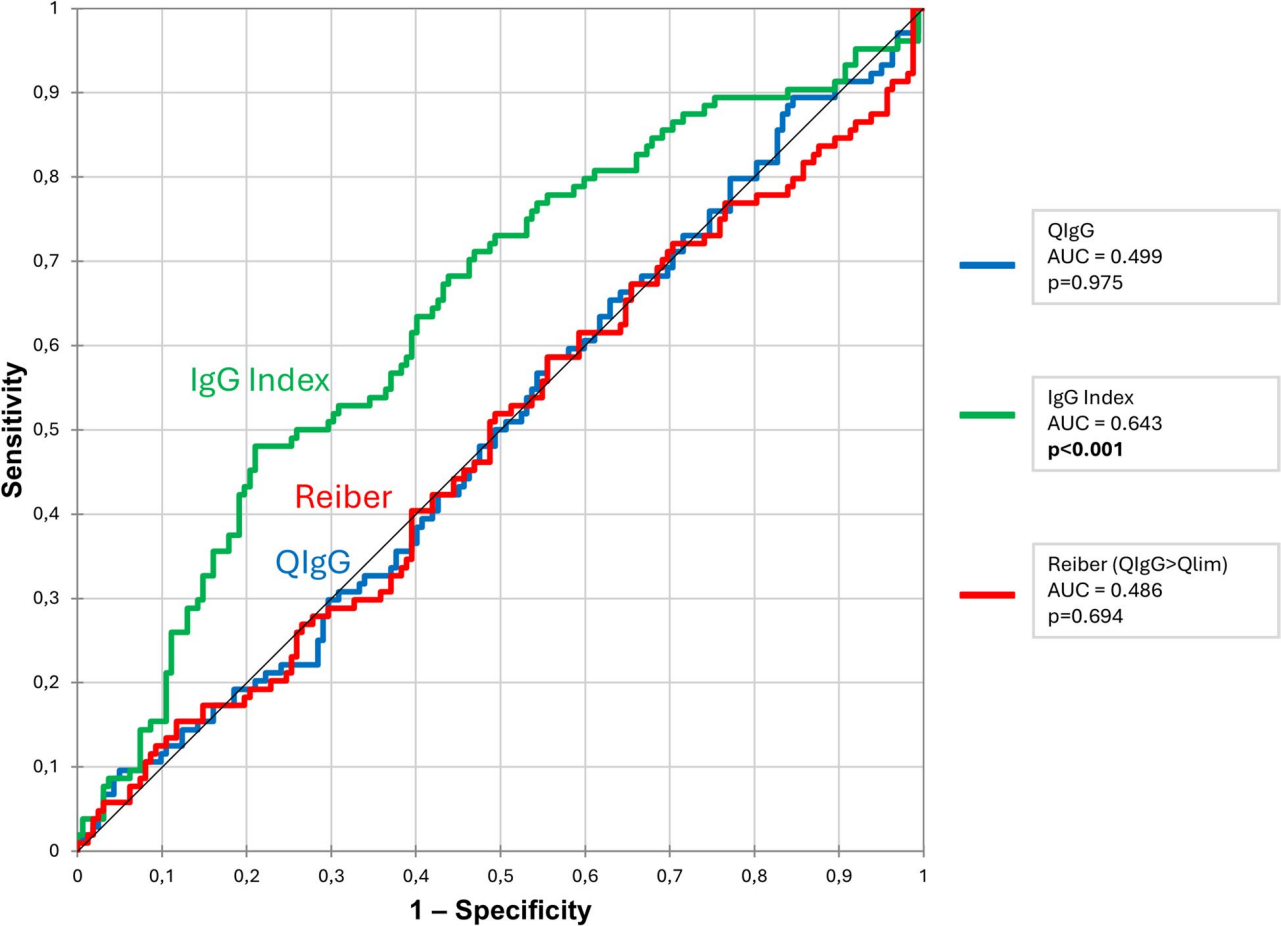
ROC curves for the prediction of CNS-AD using the 3 indexes

### Factors associated with ISI in our cohort

We then compared the factors that were significantly different between ISI positive and negative group (age, HIV, CSF leukocyte count, IgG index) in a multivariable analysis (Table 3). To avoid collinearity between overlapping CSF indexes, only the best-performing marker—IgG index—was retained for inclusion in the model. The multivariable analysis retained only age < 50 years and an IgG index > 0.7, with respective odds ratios at 2.5 [95% CI 1.3–4.7] and 14.2 [95% CI 6.6–32.0].

**Table 3:** Univariable and multivariable analyses of factors associated with ISI among the 266 patients

Factors	Univariable analysis		Multivariable analysis	
	OR [95%CI]	*p* value	OR [95%CI]	*p* value
Age < 50 years	2.7 [1.6–4.6]	< 0.0001	**2.5 [1.3–4.7]**	**0.005**
HIV positive	20.7 [3.7–117.0]	< 0.0001		
CSF leukocyte > 4/mm3	2.4 [1.4–4.0]	< 0.0001		
IgG Index > 0.7	19.5 [9.4–40.2]	< 0.0001	**14.2 [6.6–32.0]**	** < 0.001**

*CSF* Cerebro-Spinal Fluid, *HIV* Human Immunodeficient Virus, *IgG* Immunoglobulin G, *ISI* Intrathecal Synthesis of Immunoglobulins, *OR* odd ratio, *CI* confidence interval

### Comparison of “CNS-AD” patients with “Other diagnosis” patients

To evaluate the diagnostic relevance of CSF index scores for identifying CNS-AD, patients were categorized according to their final diagnosis: 104 patients (39%) were classified as CNS-AD, and 162 (61%) as “other diagnoses” (Table 4). CNS-AD patients were significantly younger than those in the “other” group, with a median age of 34 years [IQR: 25–55] versus 55 years [IQR: 39–66] (p < 0.001). CNS-AD patients also had a lower prevalence of immunodepression (6% vs. 16%, p = 0.012) due to a lower prevalence of hemopathy (2% vs. 9%, p = 0.025). Leukocyte counts in the CSF were significantly higher among CNS-AD patients compared to the “other diagnosis” group: 8/mm^3^ [IQR: 2–28] vs. 2/mm^3^ [IQR: 0–9] (p < 0.001). Among the three CSF index scores evaluated, only the IgG index showed a statistically significant difference between groups: it was positive in 35% (n = 37/104) of CNS-AD patients, compared to 16% (n = 27/162) in the “other” group (p < 0.001). Neither QIgG > 0.0035 (p = 0.944) nor Reiber’s formula > QLim (p = 0.732) differed significantly between the groups.

**Table 4:** Characteristics of the 266 patients with complete data and comparison of CSF index scores between the CNS-AD group and the “other diagnosis” group

Characteristics	Full population n = 266	CNS-AD n (%) = 104 (39)	Other diagnosis n (%) = 162 (61)	p-value
Age, years	50 [32–63]	34 [25–55]	55 [39–66]	** < 0.001**
Sex. female	113 (42)	52 (50)	61 (38)	**0.047**
Immunodepression	32 (12)	6 (6)	26 (16)	**0.012**
Solid cancer	2 (1)	0 (0)	2 (1)	0.255
Hemopathy	16 (6)	2 (2)	14 (9)	**0.025**
Transplantation	5 (2)	1 (1)	4 (2)	0.377
HIV	11 (1)	3 (3)	8 (5)	0.412
SAPS2 II at ICU admission	26 [13–44]	24 [13–38]	27 [13–45]	0.179
Mechanical ventilation (invasive)	110 (41)	50 (48	60 (37)	0.074
Length of mechanical ventilation (days)	32 [12–53]	40 [26–70]	20 [8–41]	
Length of stay in ICU (days)	15 [6–37]	28 [11–50]	12 [5–26]	
Death in ICU	24 (9)	11 (11)	13 (8)	0.478
Time between onset and ISI workup (days)	20 [7–73]	28 [12–129]	17 [4–63]	**0.014**
LP findings during ISI workup *				
Leukocytes (/mm3))	4 [1–17]	8 [2–28]	2 [0–9]	** < 0.001**
Erythrocytes (/mm3)	6 [1–75]	5 [1–53]	6 [1–84]	0.208
Protein (g/l)	0 [0–1]	0 [0–1]	0 [0–1]	0.435
Glucose (mmol/l)	4 [3, 4]	4 [3, 4]	4 [3, 4]	0.747
Albumin level (mg/l)	261 [165–447]	256 [166–385]	273 [165–484]	0.200
IgG level (mg/l)	11 [8–15]	11 [8–17]	11 [8–14]	0.594
Blood sample during ISI workup				
Albumin level (g/l)	36 [28–43]	38 [32–43]	35 [27–42]	0.062
IgG level (g/l)	50 [29–91]	51 [30–85]	48 [29–96]	0.228
ISI	93 (35)	55 (53)	38 (23)	** < 0.001**
CSF index scores				
QIgG > 0.0035	163 (61)	64 (61)	99 (61)	0.944
IgG index > 0.7	64 (24)	37 (35)	27 (16)	** < 0.001**
Reiber’s formula > 0 **	150 (56)	60 (58)	90 (55)	0.732

Results are expressed as n (%) or median [25 th;75 th percentile]. *ICU* Intensive Care Unit, *ISI* intrathecal Synthesis of Immunoglobulins, *CSF* Cerebro Spinal Fluid, *CNS* Central Nervous System, *AD* Autoimmune Disease, *HIV* Human Immunodeficient Virus, *LP* Lumbar puncture, *SAPS* Simplified Acute Physiology Score, *IgG* Immunoglobulin G, *QIgG* Quotient IgG, *QLim* QIgG upper Limit

^*^Date of first concomitant serum and LP Albumin/IgG result accessible, many patients had a prior standard LP

^**^Rebeir’s formula > 0 corresponds to QIgG > QLim

The sensitivity (Se) and specificity (Sp) for each index were calculated to assess their diagnostic performance in identifying CNS-AD (Supplementary Table 5). For QIgG > 0.0035, Se was 39% [95% CI 32–47] and Sp was 61% [95% CI 52–70]; for IgG index > 0.7, Se was 36% [95% CI 27–45] and Sp 83% [95% CI 77–88]; for Reiber’s formula (QIgG > QLim), Se was 58% [95% CI 48–67] and Sp 44% [95% CI 37–52].

Positive Predictive Values (PPV) and Negative Predictive Values (NPV) were also calculated: for QIgG > 0.0035, PPV was 62% [95% CI 52–71] and NPV 39% [95% CI 31–46]; for IgG index > 0.7, PPV was 58% [95% CI 46–70] and NPV 67% [95% CI 60–73]; and for Reiber’s formula, PPV was 40% [95% CI 32–48] and NPV 62% [95% CI 53–71]. The relationship between sensitivity and 1-specificity is illustrated in the ROC curves shown in Fig. 2.

To refine the diagnostic thresholds for predicting CNS autoimmune diseases (CNS-AD), we calculated the Youden Index for each of the three CSF indexes. This analysis, illustrated in Supplementary Fig. 3, yielded new optimal cut-off values: QIgG > 0.002, IgG index > 0.62, and Reiber’s formula > QLim + 87.5. Using these thresholds, we reassessed the cohort to compare CNS-AD and other diagnoses (Supplementary Table 6). Among the three markers, only the IgG index > 0.62 was significantly associated with CNS-AD, being present in 60% of CNS-AD cases versus 9% in the other group (p < 0.001; OR 3.4 [95% CI 2–5.7]). In contrast, neither the revised QIgG threshold (p = 0.388) nor the adjusted Reiber’s formula (p = 0.480) significantly discriminated between the two groups.

### Refinement of indexes thresholds for CNS-AD prediction

Based on the Youden Index, we defined the lower bound of a diagnostic grey zone for each CSF index, below which the diagnosis of CNS-AD was considered unlikely. To define an upper threshold, we explored the first value associated with a LR +  > 10. However, as shown in Supplementary Fig. 3, none of the three indexes reached this level of diagnostic accuracy. Consequently, no index reached a threshold with strong confirmatory value (LR +  > 10) for CNS-AD prediction in our cohort.

### Factors associated with CNS-AD diagnosis in our cohort

We then compared the factors that were significantly different between patients with a final diagnosis of CNS-AD and those with other diagnoses (Table 5). To avoid collinearity between overlapping CSF indexes, only the best-performing marker—IgG index—was retained for inclusion in the model. The multivariable analysis identified three independent factors associated with a CNS-AD diagnosis: age < 50 years (OR 2.8 [95% CI 1.6–4.8], p < 0.001), CSF leukocyte count > 4/mm^3^ (OR 2.4 [1.4–4.1], p = 0.002), and an IgG index > 0.62 (OR 2.0 [1.1–3.7], p = 0.020).

**Table 5:** Univariable and multivariable analyses of factors associated with CNS-AD diagnosis among the 266 patients

Factors	Univariable analysis		Multivariable analysis	
	OR [95%CI]	*p* value	OR [95%CI]	*p* value
Sex, female	1.6 [1–2.7]	< 0.0001		
Age < 50 years	**3 [1.8–5.1]**	** < 0.0001**	**2.8 [1.6–4.8]**	** < 0.001**
No hemopathy	4.8 [1.2–18.9]	0.025		
CSF leukocyte > 4/mm3	**2.5 [1.5–4.1]**	** < 0.0001**	**2.4 [1.4–4.1]**	**0.002**
IgG Index > 0.62	**3.4 [2–5.7]**	** < 0.001**	**2 [1.1–3.7]**	**0.020**

*CSF* Cerebro-Spinal Fluid, *HIV* Human Immunodeficient Virus, *IgG* Immunoglobulin G, *CNS-AD* Central nervous system autoimmune disease, *OR* odd ratio, *CI* confidence interval

**Fig. 3:**
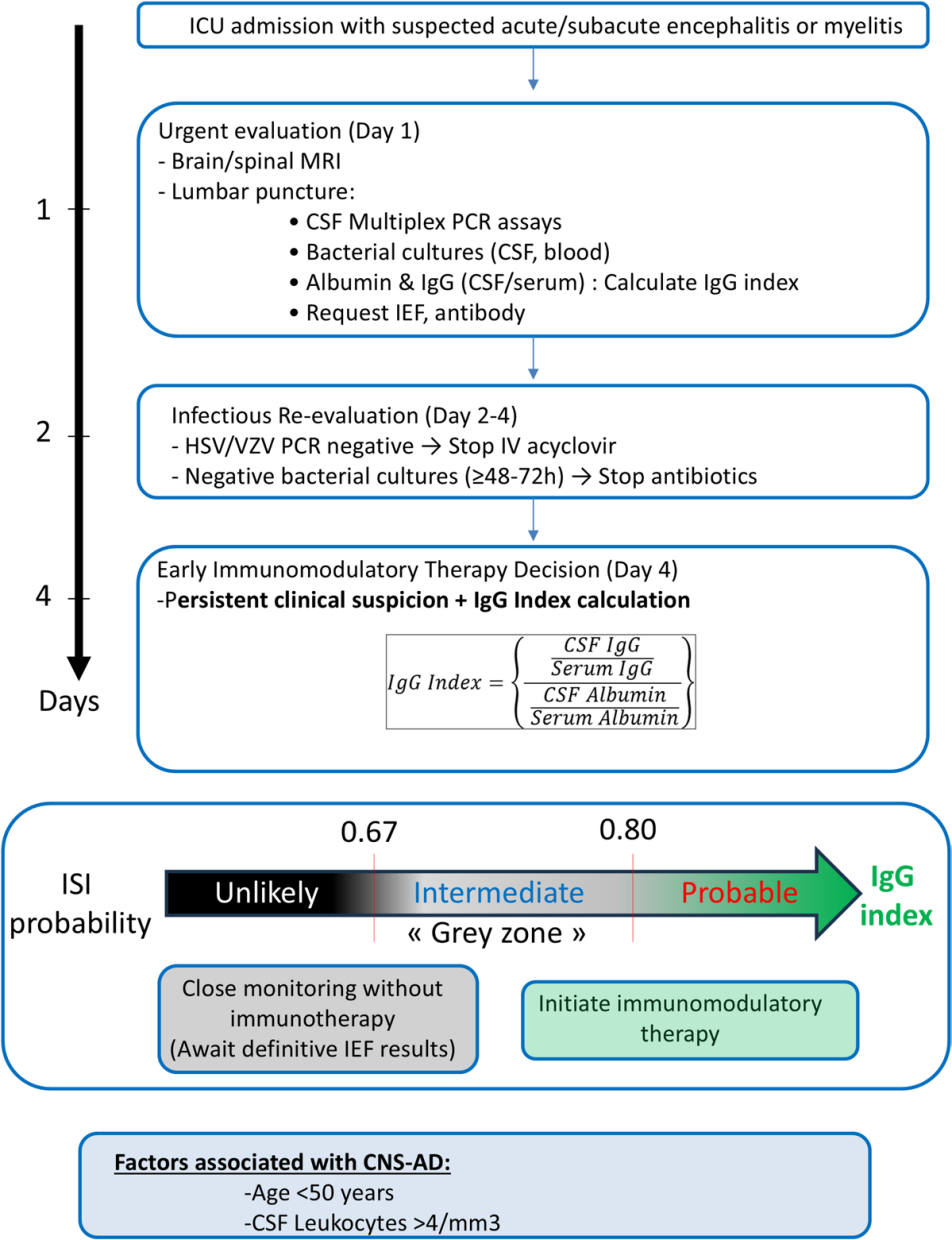
Decision algorithm integrating IgG index and clinical context

A proposed diagnostic algorithm to support clinical decision-making, including treatment considerations based on IgG index interpretation and other key clinical findings, is presented in Fig. 3.

## Discussion

Here we present the first study evaluating the diagnostic value of various CSF indexes in ICU patients using serum/CSF albumin and IgG values to predict ISI. Overall, these indexes do not perform as well in ICU patients as in the general neurological population for which they were initially validated. Among the main surrogates for IEF, the IgG index was found to be the best predictor of ISI in ICU patients.

In non-ICU patients, Reiber’s formula is usually used to predict ISI. In the literature, it has been reported to have a PPV > 99% for predicting ISI in patients with Multiple Sclerosis (MS) [10], compared to 63% in our ICU population with various diagnose. However, we did not find any statistical differences regarding this index when comparing ISI-positive and ISI-negative groups. The IgG index has also been reported to have a PPV > 99% for predict ISI in patients with MS [23], compared to 87% in our ICU population, and we found that this score, with patients age < 50, were statistically significant in multivariate analysis comparing ISI-positive and ISI-negative patients.

The high specificity of the IgG index found for ISI detection in our population (measured at 95%) and the PPV (measured at 87%), make this index useful when positive, and it could influence the decision-making process on whether to treat a patient with suspected CNS-AD while awaiting IEF results during the acute phase. However, due to its low sensitivity (measured at 56%), a negative IgG index does not exclude ISI, and clinicians should await definitive IEF results before concluding. Furthermore, clinical data, patient’s history and other complementary exams should be taken into account as ISI may indicate localized CNS inflammation rather than an autoimmune mechanism. Previous studies have focused on this question and detailed the decision making process in order to diagnose CNS-AD [24].

The lower sensitivity of these scores in our patients compared to MS patients, for whom these scores are typically used, may be a consequence of hypoalbuminemia, which is common in ICU patients [14, 15] and was also observed in our cohort.

Compared to most studies focused on MS patients, our cohort had a much more heterogeneous set of diagnoses, representative of a neurological ICU. Among these conditions, ISI was found in both CNS-AD conditions (antibody-mediated AE, CNS demyelinating inflammatory diseases, and AD with CNS involvement) but also in non-CNS-AD conditions (infectious encephalitis, tumoral involvement of the CNS, etc.…). Furthermore, the patients with MS in our study represent a specific subpopulation as most MS patients are not admitted to a neurological ICU. Therefore, the prevalence of ISI found in this population and in the context of ICU is not applicable to the general MS population.

In the literature, ISI incidence among CNS-AD is highly variable. For instance, its frequency has been reported as 72% in anti-NMDAr encephalitis [25], 65% during ADEM, 10% during neuromyelitis optica [26], 3% during MOG associated encephalomyelitis [27], and 12% in systemic AD with CNS involvement [25]. Some differences with these incidence observed in our study (Supplementary Table 7) might be a result of the specific population of this study, with patients exclusively hospitalized in a specialized neurological ICU and an up-to-date immunology laboratory, the generalizability of this prevalence to other ICUs is limited.

Interestingly, ISI presence in patients with viral encephalitis was relatively high in our cohort, although the sample size for this population was limited (Table 1). ISI was detected in 3 out of 4 cases of VZV encephalitis, and 2 out of 2 cases of HSV-2 encephalitis, suggesting a probable underlying dysimmune mechanism in these conditions. Similarly, in patients with JC virus encephalitis, 2 out of 2 tested were ISI positive, further supporting this hypothesis. In contrast, no ISI was found in the two patients with HSV-1 encephalitis and the two patients with HHV6 encephalitis who were tested. HIV-associated encephalitis showed ISI positivity in 3 out of 3 tested cases, and HIV status emerged as a risk factor for ISI positivity in univariate analysis, although it was not significant in multivariate analysis. Interestingly, none of the 10 patients with COVID-19-related encephalitic manifestations were ISI positive. While our findings are limited by the small sample sizes in each subgroup, they highlight potential differences in the pathophysiological mechanisms underlying ISI production across various form of viral encephalitis, particularly the potential role of dysimmune processes in VZV, HSV-2, and JC virus encephalitis.

Hence, in real-life clinical practice, after ruling out herpesviruses using multiplex PCR—which provides results on the same day as the LP—and obtaining the rapid HIV test result, the presence of a positive IgG index can help guide the diagnosis toward an autoimmune cause. However, it is important to emphasize that a positive IgG index does not exclude an ongoing viral dysimmune process. For instance, HIV-associated encephalitis and progressive multifocal leukoencephalopathy (PML) caused by JC virus are both conditions in which dysimmune mechanisms may play a role alongside viral replication. This highlights the need for careful interpretation of IgG index results in the broader clinical and biological context, particularly in critically ill patients, to avoid premature conclusions and ensure appropriate diagnostic and therapeutic management.

To further refine the clinical interpretation of the IgG index, we implemented a ‘grey zone’ approach based on Youden’s index and likelihood ratios. This strategy aimed to balance diagnostic accuracy with the risk of misclassification, particularly in contexts where premature immunotherapy may be harmful, such as viral encephalitis. The lower boundary of the grey zone was defined using the optimal threshold derived from the Youden index (0.67), while the upper boundary corresponded to the first value associated with a positive likelihood ratio (LR +) above 10 (0.80). As a result, IgG index values < 0.67 were considered unlikely to reflect ISI, values between 0.67 and 0.80 fell into an intermediate, uncertain zone, and values > 0.80 were interpreted as likely indicating intrathecal synthesis. This framework supports more nuanced clinical decision-making, advocating caution and confirmatory testing in borderline cases, while allowing earlier intervention in clearly positive profiles.

Importantly, none of the three indexes—QIgG, IgG index, or Reiber’s formula—achieved both high sensitivity and specificity for the identification of CNS-AD in our cohort. While these scores demonstrated acceptable diagnostic performance for predicting ISI, their ability to discriminate CNS autoimmune diseases from other conditions remained limited. This observation is consistent with the fact that these indices were not originally developed to diagnose CNS-AD, but rather to detect ISI as a marker of CNS immune activation. Our findings reinforce this limitation.

Some limitations of our study are important to consider. First, this study is retrospective and observational and therefore contains intrinsic biases. Furthermore, the monocentric aspect of conducting this study in an expert center is important to consider, as the efficiency of IEF to detect ISI might be decreased in non-expert centers [28]. Also, due to the long inclusion period, some identification and diagnostic methods have improved with time, explaining why some patients were diagnosed with anti-VGKC encephalitis at the beginning of the study, and others with anti-LG1 or anti-Caspr2 antibodies later. Furthermore, we focused on IgG indexes and did not take into account intrathecal synthesis of IgA or IgM [29]. Also, because of the limited number of subjects, the statistical analyses performed should be interpreted with caution.

Finally, as we decided to choose in our analysis the first LP with full data available, the time between disease onset and blood/CSF sampling was heterogeneous within each group itself. Moreover, some patients included in our studies had a known CNS-AD diagnosis and their blood/sampling was performed during an inflammatory flare-up of their disease, distantly from the diagnosis phase, or after treatment initiation. More generally, the decision to perform ISI testing was left to the discretion of the treating clinician and may have been influenced by prior clinical, imaging, or CSF findings. This introduces a selection bias in our cohort, which may limit the generalizability of our results.

These factors certainly had an influence on our results, as it has been described that ISI presence during neuro-Behçet’s disease, although rare, can be washed out by corticosteroids [30]. Also, CNS-AD are not a homogeneous group and prevalence of ISI vary depending on the condition, such as the identified antibody in autoimmune encephalitis. ISI reflects intrathecal inflammation, which may result from various CNS conditions, not exclusively autoimmune disorders. Treatment decisions should integrate clinical context, disease history, and complementary diagnostic findings.

As ISI presence might influence the initial therapeutic strategy in ICU, it is important to recall that IEF is the gold standard exam to confirm it, and that a multimodal approach for diagnosis orientation should still be used, taking into account the specificities of ICU patients. The technique for direct ISI identification is a comparative IEF analysis between a patient’s CSF and serum [31–33]. The presence of OCB exclusively in the patient’s CSF WB (*i.e*., absent in serum) confirms ISI [4]. However, in current practice, it can take several days to get the OCB result. Moreover, this technique, being expensive and time consuming, is typically conducted in specialized in neuroimmunology laboratories, and many centers therefore use surrogate methods for indirectly inferring ISI. Thus, we propose that the IgG index should be part of this multimodal diagnosis approach, particularly when IEF is not available, or takes too long to be returned. Unfortunately, as our study was retrospective, we were unable to assess the weight of ISI presence in the decision-making process for immunomodulatory treatment initiation.

Other scores are also being developed, such as the Kappa index [34], and these scores could have a better diagnostic performance in our population. By default, the IgG index should be preferred over Reiber’s formula or IgG quotient to predict ISI in neuro-critically-ill patients.

## Conclusion

Among the indirect indexes used in our study to predict ISI (e.g., QIgG, IgG index, and Reiber’s formula), the IgG index appears to be the more appropriate for use in ICU patients with neurological conditions, as an indirect method for ISI detection by IEF. However, IEF should still be preferred when available, as these indirect indexes demonstrate lower diagnostic performance than previously thought in ICU patients. The IgG index could be a useful and rapid tool during the acute phase of neurological assessment, helping to orient clinicians toward an autoimmune etiology and supporting the decision to initiate immunomodulatory therapy.

## Supplementary Information

Supplementary material 1: Figure 1. Study flowchart.

Supplementary material 2: Figure 2. Determination of optimal thresholds for ISI prediction based on Youden Index and Likelihood ratio.

Supplementary material 3: Figure 3. Determination of optimal thresholds for CNS-AD prediction based on Youden Index and Likelihood ratio

Supplementary material 4: Table 1. Characteristics of the 266 patients with complete data and comparison of CSF index scores between the CNS-AD group and the “other diagnosis” group. Table 2. Characteristics of the 156 patients who received an immunosuppressive treatment, and comparison of the temporality of ISI workup regarding treatment initiation. Table 3. Contingency table and diagnostic performance statistics for all three CSF indexes for ISI diagnosis. Table 4. Diagnostic performance of updated CSF index thresholdsfor predicting ISI. Table 5. Contingency table and diagnostic performance statistics for all three CSF indexes for CNS-AD diagnosis. Table 6. Diagnostic performance of updated CSF index thresholdsfor predicting CNS-AD. Table 7. Comparison of ISI prevalence in literature vs. present ICU Cohort.

## Data Availability

The author has full access to all the data; and have the right to publish all data separate and apart from any sponsor.

## Abbreviations

ADEM: Acute disseminated encephalomyelitis
AD: Autoimmune disease
AE: Autoimmune encephalitis
CANOMAD: Chronic ataxic neuropathy, ophthalmoplegia, immunoglobulin M paraprotein, cold agglutinins, and disialosyl antibodies
CASPR-2: Contactin-associated protein-like 2
CI: Confidence interval
CIDP: Chronic inflammatory demyelinating polyneuropathy
CNS: Central nervous system
COVID-19: Coronavirus disease 2019
CSF: Cerebrospinal fluid
DR2: Dopamine receptor 2
EBV: Epstein–Barr virus
GABA: γ-Aminobutyric acid
GAD65: Glutamic acid decarboxylase 65
GFAP: Glial fibrillary acidic protein
HHV-6: Human herpesvirus 6
HIV: Human immunodeficiency virus
HSV: Herpes simplex virus
IEF: Iso-electric focalization
IgLON5: IgLON family member 5
IQR: Inter-quartile range
ISI: Intrathecal synthesis of immunoglobulins
JCV: John Cunningham virus
LGI1: Leucine-rich glioma-inactivated protein 1
LOS: Length of stay
MOG: Myelin oligodendrocyte glycoprotein
MS: Multiple sclerosis
MV: Mechanical ventilation
NMDAr: N-methyl-D-aspartate receptor
OCB: Oligoclonal bands
PML: Progressive multifocal leukoencephalopathy
PRES: Posterior reversible encephalopathy
SAPSII (IGS2): Simplified acute physiology score
VGKC: Voltage-gated potassium channel
VZV: Varicella zoster virus
WB: Western blot
