# Supplementary figures and images for: Predicting intrathecal immunoglobulin synthesis in the ICU: a comparative study of IgG-based indexes
Source: Ann Intensive Care. 2025 Apr 30;15:60. doi: 10.1186/s13613-025-01475-7 (PMC12043554; doi:10.1186/s13613-025-01475-7)

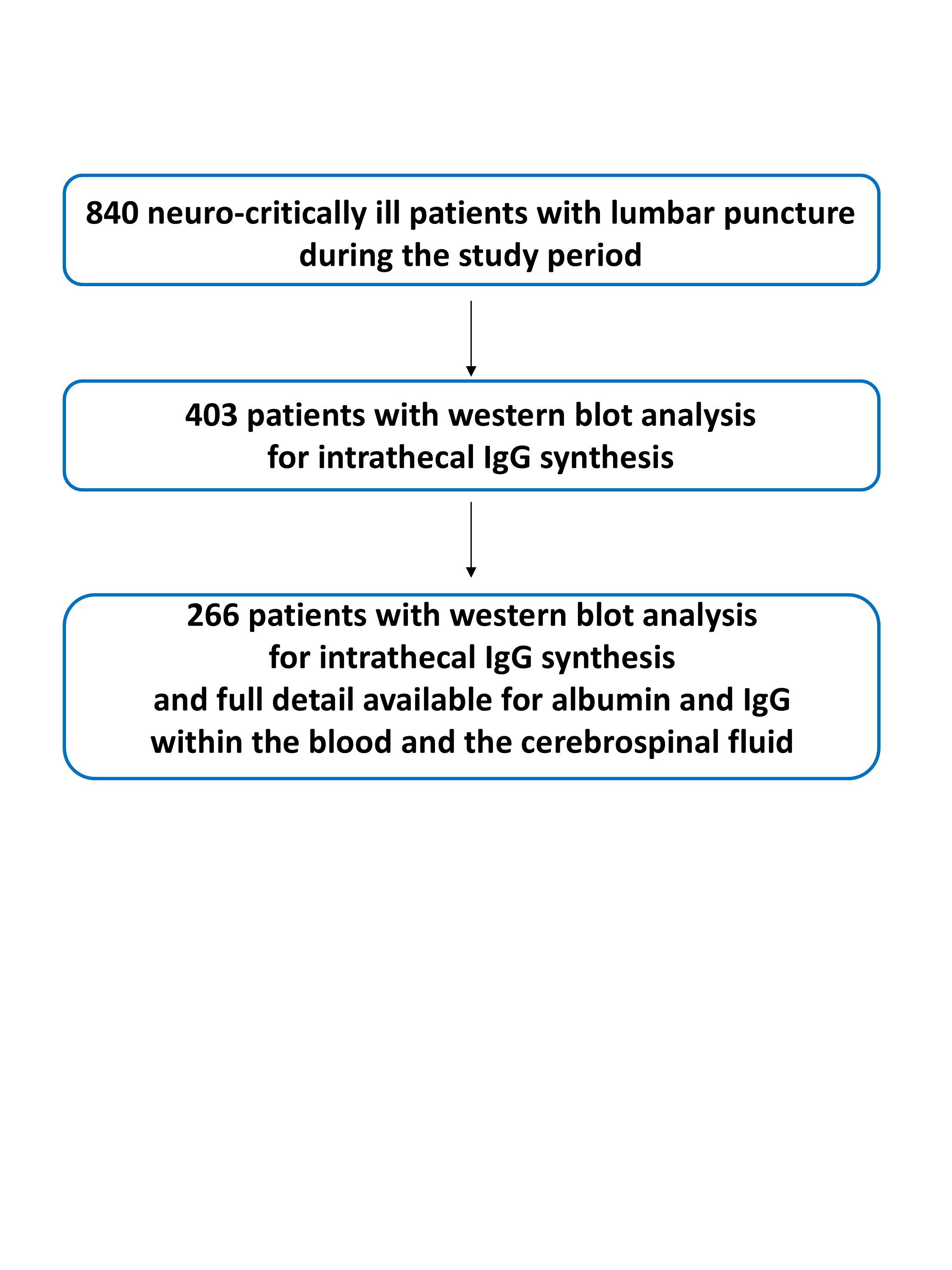

Supplement: Supplementary file 1 — Supplementary material 1: Figure 1. Study flowchart. [file 13613_2025_1475_MOESM1_ESM.bmp]

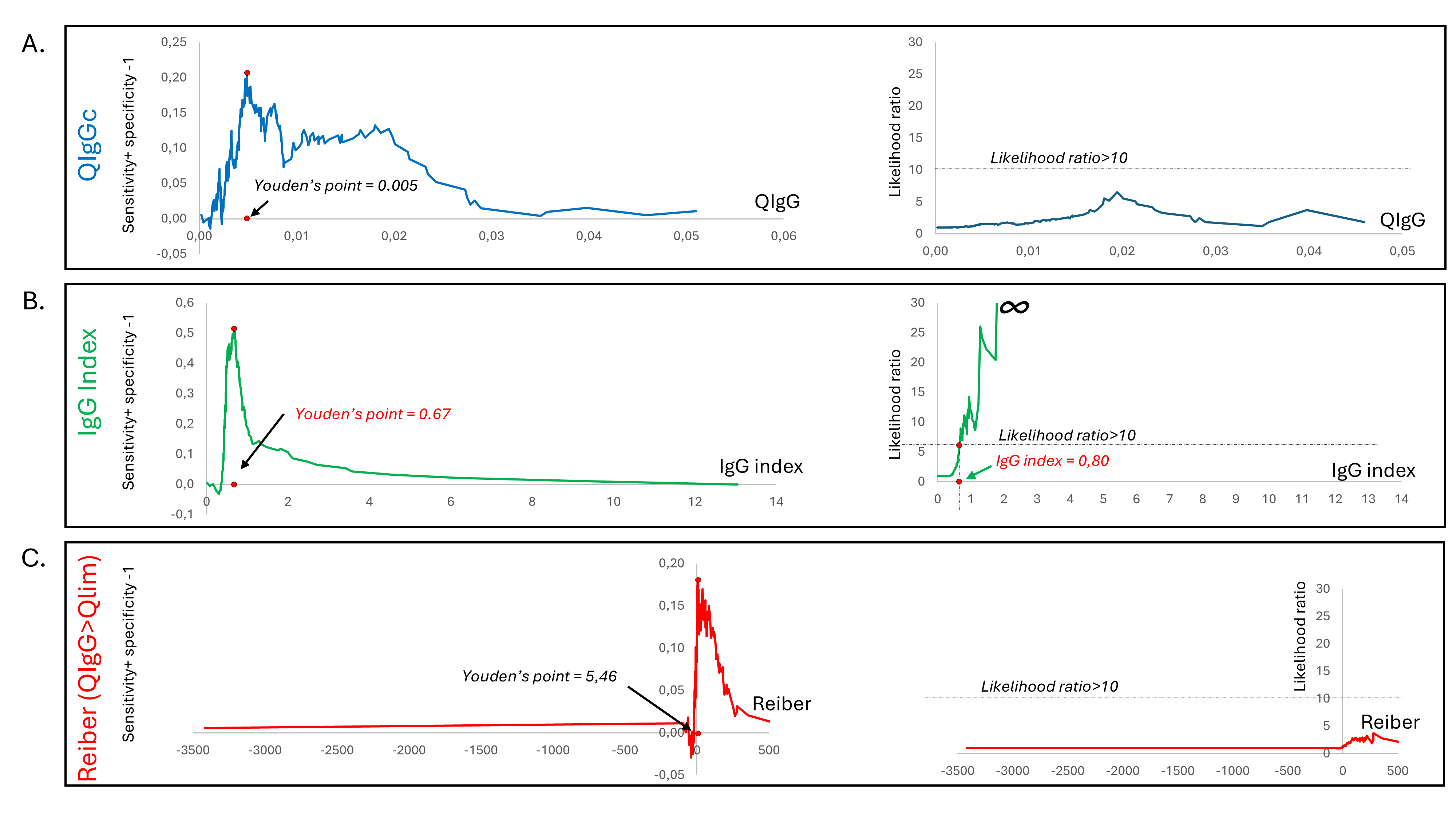

Supplement: Supplementary file 2 — Supplementary material 2: Figure 2. Determination of optimal thresholds for ISI prediction based on Youden Index and Likelihood ratio. [file 13613_2025_1475_MOESM2_ESM.bmp]

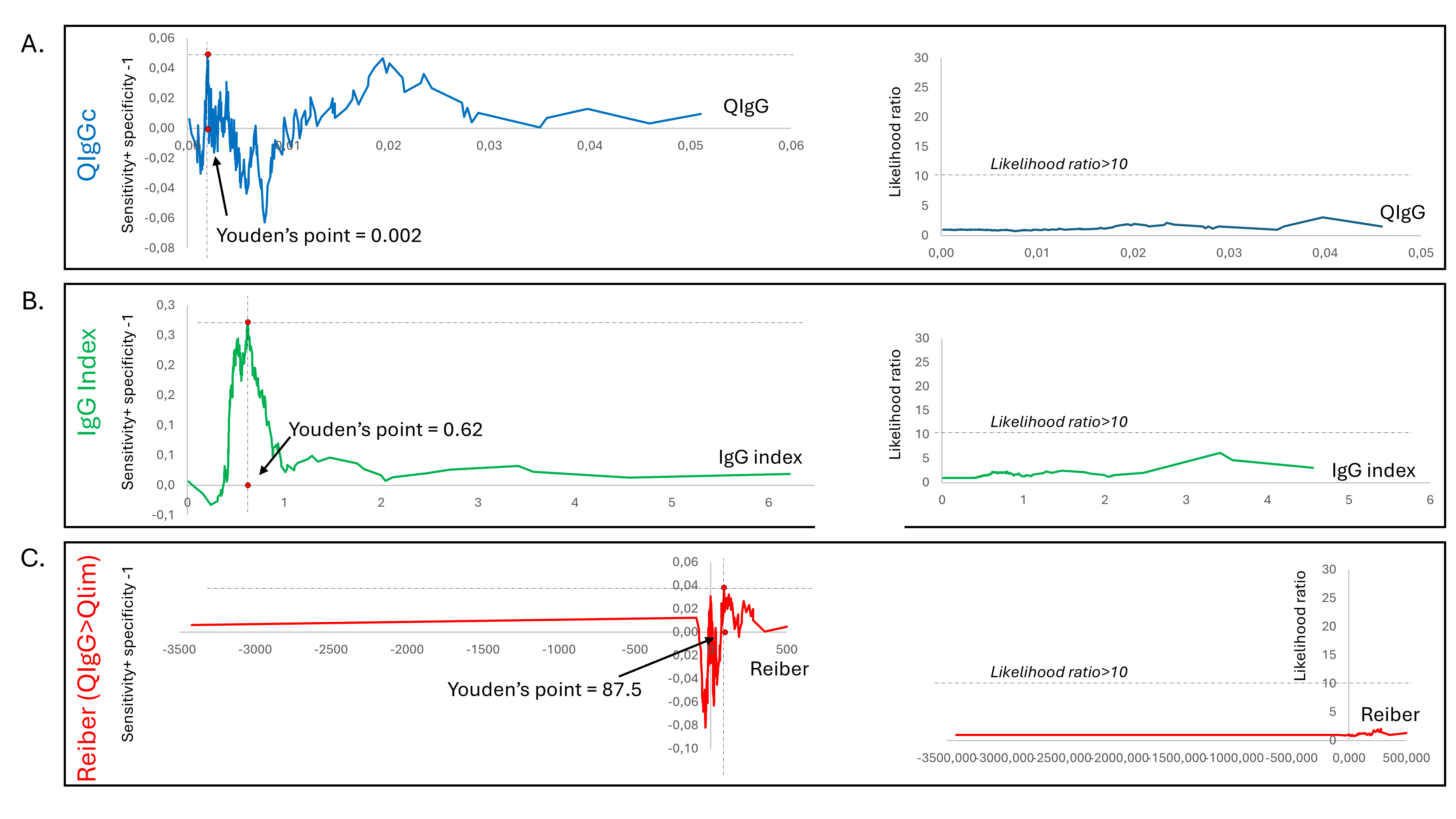

Supplement: Supplementary file 3 — Supplementary material 3: Figure 3. Determination of optimal thresholds for CNS-AD prediction based on Youden Index and Likelihood ratio [file 13613_2025_1475_MOESM3_ESM.bmp]
