# Supplementary material for: Predicting intrathecal immunoglobulin synthesis in the ICU: a comparative study of IgG-based indexes
Source: Ann Intensive Care. 2025 Apr 30;15:60. doi: 10.1186/s13613-025-01475-7 (PMC12043554; doi:10.1186/s13613-025-01475-7)
Supplement: Supplementary file 4 — Supplementary material 4: Table 1. Characteristics of the 266 patients with complete data and comparison of CSF index scores between the CNS-AD group and the “other diagnosis” group. Table 2. Characteristics of the 156 patients who received an immunosuppressive treatment, and comparison of the temporality of ISI workup regarding treatment initiation. Table 3. Contingency table and diagnostic performance statistics for all three CSF indexes for ISI diagnosis. Table 4. Diagnostic performance of updated CSF index thresholdsfor predicting ISI. Table 5. Contingency table and diagnostic performance statistics for all three CSF indexes for CNS-AD diagnosis. Table 6. Diagnostic performance of updated CSF index thresholdsfor predicting CNS-AD. Table 7. Comparison of ISI prevalence in literature vs. present ICU Cohort. [file 13613_2025_1475_MOESM4_ESM.docx]

**Supplementary table 1.** **Details of each score used in the study by final diagnosis for the 266 patients with complete data**

| **Final Diagnosis** | **Patients**  **(%)** | **ISI presence**  **n (%)** | **QIgG >0.0035**  **n (%)** | **IgGindex>0.7**  **n (%)** | **Reiber**  **n (%)** |
| --- | --- | --- | --- | --- | --- |
| **Overall population** | **266** | **93 (35)** | **169 (63)** | **61 (23)** | **151 (57)** |
| **CNS-AD** | **104 (39)** | **55 (53)** | **56 (76)** | **29 (39)** | **47 (63)** |
| **Antibody-mediated acute encephalitis** | **40 (15)** | **26 (65)** | **24 (60)** | **15 (37)** | **24 (60)** |
| Anti-NMDAr | 28 | 21 (75) | 15 (54) | 13 (46) | 14 (50) |
| Anti-GAD65 | 2 | 2 (100) | 1 (50) | 0 (0) | 1 (50) |
| Anti-LGI1 | 2 | 0 (0) | 2 (100) | 0 (0) | 2 (100) |
| Anti-CASPR-2 | 2 | 0 (0) | 1 (50) | 0 (0) | 1 (50) |
| Anti-IgLON5 | 2 | 0 (0) | 2 (100) | 0 (0) | 2 (100) |
| Anti-GFAP | 2 | 2 (100) | 2 (100) | 1 (50) | 2 (100) |
| Anti-GABA-A | 1 | 0 (0) | 1(100) | 1 (100) | 1 (100) |
| Anti-DR2 | 1 | 1 (100) | 0 (0) | 0 (0) | 1 (100) |
| **Seronegative CNS-AD** | **30 (11)** | **11 (37)** |  |  |  |
| Definite autoimmune encephalitis | 4 | 1 (25) | 1 (25) | 1 (25) | 1 (25) |
| Possible autoimmune encephalitis | 23 | 9 (39) | 12 (52) | 12 (52) | 11 (48) |
| Idiopathic acute transverse myelitis | 3 | 1 (33) | 2 (66) | 1 (33) | 2 (66) |
| **Central demyelinating inflammatory diseases** | **22 (8)** | **11 (50)** | **16 (72)** | **7 (32)** | **15 (68)** |
| Multiple sclerosis | 5 | 4 (80) | 5 (100) | 2 (40) | 4 (80) |
| ADEM | 6 | 3 (50) | 5 (83) | 2 (33) | 5 (83) |
| Neuromyelitis optica | 7 | 3 (43) | 4 (57) | 2 (29) | 4 (57) |
| MOG antibody-associated encephalomyelitis | 4 | 1 (25) | 2 (50) | 1 (25) | 2 (50) |
| **Systemic AD with CNS involvement** | **12 (4.5)** | **7 (58)** | **8 (66)** | **7 (58)** | **8 (66)** |
| Behçet | 3 | 2 (67) | 1 (33) | 1 (33) | 1 (33) |
| Sarcoidosis | 4 | 3 (75) | 3(75) | 3 (75) | 3 (75) |
| Cerebral mastocytosis | 1 | 1 (100) | 1 (100) | 1 (100) | 1 (100) |
| Lupus | 1 | 0 (0) | 1 (100) | 1 (100) | 1 (100) |
| Central nervous system vasculitis | 3 | 1 (33) | 2 (66) | 1 (33) | 2 (66) |
| **Other diagnosis** | **162 (61)** | **38 (23)** | **113 (59)** | **32 (17)** | **104 (54)** |
| **CNS affection of unknown etiology** | **38 (14)** | **14 (37)** |  |  |  |
| Encephalitis of unknown etiology | 25 | 7 (28) | 15 (60) | 5 (20) | 12 (8) |
| Myelitis of unknown etiology | 7 | 4 (57) | 6 (85) | 3 (43) | 4 (57) |
| Aseptic meningitis | 6 | 3 (50) | 6 (100) | 0 (0) | 6 (100) |
| **Cerebrovascular diseases** | **7 (2.6)** | **0 (0)** | **3 (42)** | **0 (0)** | **2 (28)** |
| PRES | 2 | 0 (0) | 1 (50) | 0 (0) | 0 (0) |
| Ischemic stroke and anoxic encephalopathy | 4 | 0 (0) | 2 (50) | 0 (0) | 2 (50) |
| Arteriovenous malformation | 1 | 0 (0) | 0 (0) | 0 (0) | 0 (0) |
| **Viral infections** | **21 (7.8)** | **8 (38)** | **11 (52)** | **7 (33)** | **11 (52)** |
| HSV-1 encephalitis | 1 | 0 (0) | 0 (0) | 0 (0) | 0 (0) |
| VZV encephalitis | 2 | 2 (100) | 2 (100) | 1 (50) | 2 (100) |
| HSV-2 encephalitis | 1 | 1 (100) | 1 (100) | 0 (0) | 1 (100) |
| HHV-6 encephalitis | 2 | 0 (0) | 2 (100) | 0 (0) | 2 (100) |
| HIV encephalitis | 3 | 3 (100) | 2 (66) | 3 (100) | 2 (66) |
| JCV encephalitis * | 2 | 2 (100) | 2 (100) | 2 (100) | 2 (100) |
| COVID-19 associated encephalitis | 10 | 0 (0) | 3 (30) | 1 (10) | 2 (20) |
| **Bacterial infections** | **9 (3.3)** | **4 (44)** | **8 (88)** | **3 (33)** | **8 (88)** |
| Q Fever meningoencephalitis | 1 | 0 (0) | 1 (100) | 0 (0) | 1 (100) |
| Tuberculous meningoencephalitis | 8 | 4 (50) | 7 (88) | 3 (38) | 7 (87) |
| **Fungal/Parasite infections** | **5 (1.8)** | **1 (25)** | **3 (60)** | **3 (60)** | **3 (60)** |
| Aspergillus | 2 | 0 (0) | 2 (100) | 2 (100) | 2 (100) |
| Neurotoxocariasis | 3 | 1 (33) | 1 (33) | 1 (33) | 1 (33) |
| **Tumoral involvement of nervous system** | **28 (10)** | **6 (75)** | **20 (71)** | **4 (14)** | **19 (68)** |
| Glioma | 9 | 0 (0) | 4 (44) | 0 (0) | 3 (33) |
| Primary CNS lymphoma | 7 | 3 (43) | 6 (85) | 2 (28) | 6 (85) |
| B-Cell systemic lymphoma | 2 | 0 (0) | 2 (100) | 0 (0) | 2 (100) |
| T-Cell systemic lymphoma | 2 | 2 (100) | 1 (50) | 1 (50) | 1 (50) |
| Hodgkin lymphoma | 1 | 0 (0) | 0 (0) | 0 (0) | 0 (0) |
| Chronic lymphocytic leukemia | 1 | 0 (0) | 1 (100) | 0 (0) | 1 (100) |
| Bing-Neel syndrome | 1 | 1 (100) | 1 (100) | 1 (100) | 1 (100) |
| Graft versus host | 1 | 0 (0) | 1 (100) | 0 (0) | 1 (100) |
| Brain metastasis of solid cancer | 4 | 0 (0) | 4 (100) | 0 (0) | 4 (100) |
| **Inflammatory Polyradiculopathies** | **17 (6.3)** | **1 (6)** | **14 (82)** | **0 (0)** | **14 (82)** |
| Guillain-Barre syndrome | 13 | 0 (0) | 11 (84) | 0 (0) | 11 (85) |
| CIDP | 3 | 0 (0) | 3 (100) | 0 (0) | 3 (100) |
| Parsonage-Turner syndrome | 1 | 1 (100) | 0 (0) | 0 (0) | 0 (0) |
| **Miscellaneous** | **37 (14)** | **4 (11)** | **12 (37)** | **1 (2)** | **11 (30)** |
| Neurodegenerative disease | 8 | 0 (0) | 0 (0) | 0 (0) | 0 (0) |
| Functional neurological disorders | 8 | 0 (0) | 0 (0) | 0 (0) | 0 (0) |
| Creutzfeldt Jakob | 4 | 1 (25) | 2 (50) | 0 (0) | 2 (50) |
| Toxic Metabolic disorders + IEM | 13 | 2 (15) | 9 (69) | 1 (8) | 8 (61) |
| Seizure (non-immune related) | 3 | 0 (0) | 1 (33) | 0 (0) | 1 (33) |
| Lambert-Eaton | 1 | 1 (100) | 0 (0) | 0 (0) | 0 (0) |

Results are expressed as number (%) or median [27th;75th percentile]. n. number of patients; Q. quotient; Alb. albumin. Lim. limit; IgG. immunoglobulin G; ICU. Intensive Care Unit; ; Acute Encephalitis; CNS, Central Nervous System; AD, autoimmune disease; OCBs. Oligoclonal Bands; IEM. Inborn error of metabolism; ADEM. Acute disseminated encephalomyelitis; MOG. Myelin oligodendrocyte glycoprotein; GBS. Guillain Barre Syndrome; CIDP. Chronic inflammatory demyelinating polyneuropathy; NMDAr. N-methyl-D-aspartate receptor; GAD65. glutamic acid decarboxylase 65; LGI1. Leucine-rich glioma-inactivated protein 1; CASPR-2. Contactin-associated protein-like 2; IgLON5. IgLON Family Member 5; GFAP. Glial fibrillary acidic protein; GABA. γ-Aminobutyric acid; DR2. Dopamine Receptor 2; ME. meningo-encephalitis; HHV-6. Human herpesvirus 6; HSV. Herpes simplex virus; VZV. Varicella zoster virus; JCV. John Cunningham virus; MS. Multiple Sclerosis; HIV. human immunodeficiency virus; COVID-19. coronavirus disease 2019; PRES. Posterior reversible encephalopathy.

*Among the two patients with JCV encephalitis: 1 had HIV, and 1 was treated with Fingolimod for MS

|  | Number of patient who received an immunosuppressive treatment  n=156 | Immunosuppressive treatment after ISI workup *  n=117 | Immunosuppressive treatment before ISI workup  n=39 | p-value |
| --- | --- | --- | --- | --- |
|  |  |  |  |  |
| CNS-AD patients | **96 (62)** | **68 (58)** | **28 (72)** |  |
| ISI positive | 50 (53) | 35 (52) | 15 (54) | 0.851 |
| Other patients | **60 (38)** | **49 (42)** | **11 (28)** |  |
| ISI positive | 13 (22) | 12 (24) | 1 (9) | 0.263 |
| Characteristics |  |  |  |  |
| SAPS2 II at ICU admission | 26 [13-42] | 28 [13-43] | 23 [12-38] | 0.319 |
| Time between onset and ISI workup (days)* | 19 [10-123] | 16 [8-63] | 53 [23-202] | **<0.001** |
| LP findings during ISI workup * |  |  |  |  |
| Leukocytes (/mm3)) | 6 [1-26] | 6 [1-28] | 6 [1-20] | 0.823 |
| Erythrocytes (/mm3) | 8 [1-46] | 6 [1-39] | 13 [1-196] | 0.189 |
| Protein (g/l) | 1 [0-1] | 1 [0-1] | 1 [0-1] | 0.491 |
| Glucose (mmol/l) | 4 [3-4] | 4 [3-4] | 4 [3-4] | 0.388 |
| Albumin level (mg/l) | 278 [186-483] | 275 [173-475] | 312 [211-502] | 0.347 |
| IgG level (mg/l) | 57 [37-109] | 58 [36-109] | 54 [39-110] | 0.985 |
| Blood sample during ISI workup |  |  |  |  |
| Albumin level (g/l) | 37 [29-42] | 36 [28-42] | 38 [33-43] | 0.149 |
| IgG level (g/l) | 12 [8-17] | 12 [9-16] | 12 [7-19] | 0.797 |
| CSF index scores |  |  |  |  |
| QIgG>0.0035 | 106 | 81 | 25 | 0.552 |
| IgG index>0.7 | 48 | 36 | 12 | 0.841 |
| Reiber’s formula>0 ** | 101 | 78 | 23 | 0.384 |

**Supplementary table 2. Characteristics of the 156 patients who received an immunosuppressive treatment, and comparison of the temporality of ISI workup regarding treatment initiation**

Results are expressed as n (%) or median [25^th^;75th percentile]. ICU, Intensive Care Unit; ISI, intrathecal Synthesis of Immunoglobulins; CSF, Cerebro Spinal Fluid; CNS, Central Nervous System; AD, Autoimmune Disease; LP, Lumbar puncture; IgG, Immunoglobulin G; QIgG, Quotient IgG. QLim, QIgG upper Limit

*Here, the association between ISI results and the decision to initiate immunosuppressive therapy is not investigated

** Rebeir’s formula>0 corresponds to QIgG>QLim

**Supplementary Table 3. Contingency table and diagnostic performance statistics for all three CSF indexes for ISI diagnosis**

A . Contingency table for QIgG>0.0035:

|  |  |  |  |
| --- | --- | --- | --- |
|  | ISI present | ISI absent |  |
| QIgG > 0.0035 | 62 | 101 |  |
| QIgG < 0.0035 | 31 | 72 |  |
|  |  |  |  |
|  |  |  |  |
| Statistics | Value | Lower 95% confidence threshold | Upper 95% confidence threshold |
| Correct classification | 0.502 | 0.442 | 0.562 |
| Incorrect classification | 0.498 | 0.438 | 0.558 |
| **Sensibility** | **0.667** | **0.566** | **0.754** |
| **Specificity** | **0.414** | **0.343** | **0.488** |
| Fraction of false positive | 0.586 | 0.514 | 0.659 |
| Fraction of false negative | 0.333 | 0.240 | 0.427 |
| Prevalence | 0.348 | 0.291 | 0.405 |
| **Positive predictive value** | **0.378** | **0.304** | **0.452** |
| **Negative predictive value** | **0.699** | **0.610** | **0.788** |
| **Positive likelihood ratio** | **1.137** | **0.940** | **1.376** |
| **Negative likelihood ratio** | **0.806** | **0.575** | **1.129** |
| Relative risk ratio | 1.256 | 0.885 | 1.784 |
| Odds ratio | 1.412 | 0.837 | 2.383 |

B . Contingency table for QIgG>0.005:

|  |  |  |  |
| --- | --- | --- | --- |
|  | ISI present | ISI absent |  |
| QIgG > 0.005 | 49 | 59 |  |
| QIgG < 0.005 | 44 | 114 |  |
|  |  |  |  |
|  |  |  |  |
| Statistics | Value | Lower 95% confidence threshold | Upper 95% confidence threshold |
| Correct classification | 0,613 | 0,554 | 0,671 |
| Incorrect classification | 0,387 | 0,329 | 0,446 |
| **Sensibility** | **0,527** | **0,426** | **0,625** |
| **Specificity** | **0,659** | **0,585** | **0,725** |
| Fraction of false positive | 0,341 | 0,271 | 0,411 |
| Fraction of false negative | 0,473 | 0,374 | 0,572 |
| Prevalence | 0,350 | 0,292 | 0,407 |
| **Positive predictive value** | 0,454 | 0,360 | 0,548 |
| **Negative predictive value** | 0,722 | 0,652 | 0,791 |
| Positive likelihood ratio | 1,545 | 1,164 | 2,050 |
| Negative likelihood ratio | 0,718 | 0,565 | 0,913 |
| Relative risk ratio | 1,629 | 1,180 | 2,250 |
| Odds ratio | 2,152 | 1,290 | 3,590 |

C .Contingency table for IgG index>0.7:

|  |  |  |  |
| --- | --- | --- | --- |
|  | ISI present | ISI absent |  |
| IgG index > 0.7 | 53 | 8 |  |
| IgG index < 0.7 | 40 | 165 |  |
|  |  |  |  |
|  |  |  |  |
| Statistics | Value | Lower 95% confidence threshold | Upper 95% confidence threshold |
| Correct classification | 0.816 | 0.770 | 0.863 |
| Incorrect classification | 0.184 | 0.137 | 0.230 |
| **Sensibility** | **0.559** | **0.458** | **0.656** |
| **Specificity** | **0.954** | **0.910** | **0.978** |
| Fraction of false positive | 0.046 | 0.015 | 0.077 |
| Fraction of false negative | 0.441 | 0.342 | 0.540 |
| Prevalence | 0.348 | 0.291 | 0.405 |
| **Positive predictive value** | **0.867** | **0.781** | **0.953** |
| **Negative predictive value** | **0.802** | **0.748** | **0.856** |
| **Positive likelihood ratio** | **12.161** | **6.036** | **24.502** |
| **Negative likelihood ratio** | **0.462** | **0.367** | **0.582** |
| Relative risk ratio | 4.376 | 3.276 | 5.844 |
| Odds ratio | 26.317 | 11.820 | 58.597 |

D .Contingency table for IgG index>0.67:

|  |  |  |  |
| --- | --- | --- | --- |
|  | ISI present | ISI absent |  |
| IgG index > 0.67 | 56 | 16 |  |
| IgG index < 0.67 | 37 | 157 |  |
|  |  |  |  |
|  |  |  |  |
| Statistics | Value | Lower 95% confidence threshold | Upper 95% confidence threshold |
| Correct classification | 0,801 | 0,753 | 0,849 |
| Incorrect classification | 0,199 | 0,151 | 0,247 |
| **Sensibility** | **0,602** | **0,500** | **0,696** |
| **Specificity** | **0,908** | **0,854** | **0,943** |
| Fraction of false positive | 0,092 | 0,050 | 0,135 |
| Fraction of false negative | 0,398 | 0,300 | 0,495 |
| Prevalence | 0,350 | 0,292 | 0,407 |
| **Positive predictive value** | **0,778** | **0,682** | **0,874** |
| **Negative predictive value** | **0,809** | **0,754** | **0,865** |
| **Positive likelihood ratio** | **6,511** | **3,968** | **10,683** |
| **Negative likelihood ratio** | **0,438** | **0,340** | **0,565** |
| Relative risk ratio | 4,078 | 2,983 | 5,574 |
| Odds ratio | 14,851 | 7,723 | 28,561 |

E .Contingency table for Reiber’s formula (QIgG>QLim):

|  | ISI present | ISI absent |  |
| --- | --- | --- | --- |
| Reiber’s formula QIgG>QLim | 59 | 91 |  |
| Reiber’s formula QIgG<QLim | 34 | 82 |  |
|  |  |  |  |
|  |  |  |  |
| Statistics | Value | Lower 95% confidence threshold | Upper 95% confidence threshold |
| Correct classification | 0.530 | 0.470 | 0.590 |
| Incorrect classification | 0.470 | 0.410 | 0.530 |
| **Sensibility** | **0.393** | **0.319** | **0.473** |
| **Specificity** | **0.707** | **0.618** | **0.782** |
| Fraction of false positive | 0.293 | 0.212 | 0.375 |
| Fraction of false negative | 0.607 | 0.530 | 0.684 |
| Prevalence | 0.564 | 0.504 | 0.624 |
| **Positive predictive value** | **0.634** | **0.537** | **0.732** |
| **Negative predictive value** | **0.474** | **0.400** | **0.548** |
| **Positive likelihood ratio** | **1.342** | **0.950** | **1.896** |
| **Negative likelihood ratio** | **0.858** | **0.721** | **1.021** |
| Relative risk ratio | 1.206 | 0.979 | 1.485 |
| Odds ratio | 1.564 | 0.935 | 2.615 |

F .Contingency table for QIgG>QLim + 5.46

|  | ISI present | ISI absent |  |
| --- | --- | --- | --- |
| QIgG>QLim + 5.46 | 56 | 74 |  |
| QIgG>QLim + 5.46 | 37 | 99 |  |
|  |  |  |  |
|  |  |  |  |
| Statistics | Value | Lower 95% confidence threshold | Upper 95% confidence threshold |
| Correct classification | 0,583 | 0,523 | 0,642 |
| Incorrect classification | 0,417 | 0,358 | 0,477 |
| **Sensibility** | **0,602** | **0,500** | **0,696** |
| **Specificity** | **0,572** | **0,498** | **0,644** |
| Fraction of false positive | 0,428 | 0,355 | 0,501 |
| Fraction of false negative | 0,398 | 0,300 | 0,495 |
| Prevalence | 0,350 | 0,292 | 0,407 |
| **Positive predictive value** | **0,431** | **0,346** | **0,516** |
| **Negative predictive value** | **0,728** | **0,653** | **0,803** |
| **Positive likelihood ratio** | **1,408** | **1,109** | **1,787** |
| **Negative likelihood ratio** | **0,695** | **0,525** | **0,921** |
| Relative risk ratio | 1,583 | 1,132 | 2,216 |
| Odds ratio | 2,025 | 1,215 | 3,374 |

Abbreviations: Q. quotient; IgG. Immunoglobulin G; ISI. Intrathecal Synthesis of Immunoglobulins

QIgG, Quotient IgG. QLim, QIgG upper Limit

| Characteristics | Full population  n=266 | ISI-positive  patients  n (%) =93 (35) | ISI-negative  patients  n (%) =173 (65) | OR [95%CI] | p-value |
| --- | --- | --- | --- | --- | --- |
| CSF Index scores (Table 2) |  |  |  |  |  |
| QIgG>0.0035 | 162 (62) | 62 (67) | 101 (59) | 1.4 [0.8-2.4] | 0.218 |
| IgG index>0.7 | 64 (24) | 53 (57) | 11 (6) | **19.5 [9.4-40.2]** | **<0.001** |
| Reiber’s formula>0 * | 150 (56) | 59 (63) | 91 (34) | 1.5 [0.9-2.6] | 0.089 |
| New index’s cut-off |  |  |  |  |  |
| QIgG>0.005 | 108 (40) | 49 (52) | 59 (34) | **2.1 [1.3-3.6]** | **0.003** |
| IgG index>0.67 | 72 (27) | 56 (60) | 16 (9) | **14.8 [7.7-28.5]** | **<0.001** |
| QIgG>QLim + 5.46 | 130 (49) | 56 (60) | 74 (42) | **2 [1.2-3.3]** | **0.007** |

**Supplementary table 3. Comparison of CSF index scores regarding ISI with new index’s cut-off scores**

Results are expressed as n (%)

Abbreviations: ISI, intrathecal Synthesis of Immunoglobulins; CSF, Cerebro Spinal Fluid; IgG, Immunoglobulin G; QIgG, Quotient IgG. QLim, QIgG upper Limit

* Reiber’s formula>0 corresponds to QIgG>QLim

**Supplementary Table 5. Contingency table and diagnostic performance statistics for all three CSF indexes for CNS-AD diagnosis**

A . Contingency table for QIgG>0.0035:

|  |  |  |  |
| --- | --- | --- | --- |
|  | CNS-AD | Other |  |
| QIgG > 0.0035 | 64 | 99 |  |
| QIgG < 0.0035 | 40 | 63 |  |
|  |  |  |  |
|  |  |  |  |
| Statistics | Value | Lower 95% confidence threshold | Upper 95% confidence threshold |
| Correct classification | 0.477 | 0.417 | 0.537 |
| Incorrect classification | 0.523 | 0.463 | 0.583 |
| **Sensibility** | **0.393** | **0.321** | **0.469** |
| **Specificity** | **0.612** | **0.515** | **0.700** |
| Fraction of false positive | 0.388 | 0.296 | 0.481 |
| Fraction of false negative | 0.607 | 0.533 | 0.681 |
| Prevalence | 0.613 | 0.554 | 0.671 |
| **Positive predictive value** | **0.615** | **0.522** | **0.709** |
| **Negative predictive value** | **0.389** | **0.314** | **0.464** |
| **Positive likelihood ratio** | **1.011** | **0.743** | **1.376** |
| **Negative likelihood ratio** | **0.993** | **0.815** | **1.210** |
| Relative risk ratio | 1.007 | 0.829 | 1.223 |
| Odds ratio | 1.018 | 0.615 | 1.685 |

B . Contingency table for QIgG>0.002:

|  |  |  |  |
| --- | --- | --- | --- |
|  | CNS-AD | Other |  |
| QIgG > 0.002 | 93 | 139 |  |
| QIgG < 0.002 | 11 | 23 |  |
|  |  |  |  |
|  |  |  |  |
| Statistics | Value | Lower 95% confidence threshold | Upper 95% confidence threshold |
| Correct classification | 0.436 | 0.376 | 0.496 |
| Incorrect classification | 0.564 | 0.504 | 0.624 |
| **Sensibility** | **0.894** | **0.818** | **0.941** |
| **Specificity** | **0.142** | **0.096** | **0.205** |
| Fraction of false positive | 0.858 | 0.805 | 0.911 |
| Fraction of false negative | 0.106 | 0.048 | 0.164 |
| Prevalence | 0.391 | 0.332 | 0.450 |
| **Positive predictive value** | **0.401** | **0.338** | **0.464** |
| **Negative predictive value** | **0.676** | **0.519** | **0.834** |
| **Positive likelihood ratio** | **1.042** | **0.951** | **1.142** |
| **Negative likelihood ratio** | **0.745** | **0.379** | **1.463** |
| Relative risk ratio | 1.239 | 0.754 | 2.037 |
| Odds ratio | 1.399 | 0.659 | 2.969 |

C .Contingency table for IgG index>0.7:

|  |  |  |  |
| --- | --- | --- | --- |
|  | CNS-AD | Other |  |
| IgG index > 0.7 | 37 | 27 |  |
| IgG index < 0.7 | 67 | 135 |  |
|  |  |  |  |
|  |  |  |  |
| Statistics | Value | Lower 95% confidence threshold | Upper 95% confidence threshold |
| Correct classification | 0.647 | 0.589 | 0.704 |
| Incorrect classification | 0.353 | 0.296 | 0.411 |
| **Sensibility** | **0.356** | **0.271** | **0.452** |
| **Specificity** | **0.833** | **0.768** | **0.883** |
| Fraction of false positive | 0.167 | 0.110 | 0.223 |
| Fraction of false negative | 0.644 | 0.554 | 0.735 |
| Prevalence | 0.391 | 0.332 | 0.450 |
| **Positive predictive value** | **0.578** | **0.457** | **0.699** |
| **Negative predictive value** | **0.668** | **0.603** | **0.733** |
| **Positive likelihood ratio** | **2.135** | **1.388** | **3.284** |
| **Negative likelihood ratio** | **0.773** | **0.660** | **0.906** |
| Relative risk ratio | 1.743 | 1.312 | 2.316 |
| Odds ratio | 2.761 | 1.558 | 4.893 |

D .Contingency table for IgG index>0.62:

|  |  |  |  |
| --- | --- | --- | --- |
|  | CNS-AD | Other |  |
| IgG index > 0.62 | 50 | 35 |  |
| IgG index < 0.62 | 54 | 127 |  |
|  |  |  |  |
|  |  |  |  |
| Statistics | Value | Lower 95% confidence threshold | Upper 95% confidence threshold |
| Correct classification | 0.665 | 0.609 | 0.722 |
| Incorrect classification | 0.335 | 0.278 | 0.391 |
| **Sensibility** | **0.481** | **0.387** | **0.576** |
| **Specificity** | **0.784** | **0.714** | **0.840** |
| Fraction of false positive | 0.216 | 0.153 | 0.279 |
| Fraction of false negative | 0.519 | 0.425 | 0.613 |
| Prevalence | 0.391 | 0.332 | 0.450 |
| **Positive predictive value** | **0.588** | **0.484** | **0.693** |
| **Negative predictive value** | **0.702** | **0.635** | **0.768** |
| **Positive likelihood ratio** | **2.225** | **1.561** | **3.173** |
| **Negative likelihood ratio** | **0.662** | **0.541** | **0.810** |
| Relative risk ratio | 1.972 | 1.485 | 2.618 |
| Odds ratio | 3.360 | 1.970 | 5.730 |

E .Contingency table for Reiber’s formula (QIgG>QLim):

|  | CNS-AD | Other |  |
| --- | --- | --- | --- |
| Reiber’s formula QIgG>QLim | 60 | 90 |  |
| Reiber’s formula QIgG<QLim | 44 | 72 |  |
|  |  |  |  |
|  |  |  |  |
| Statistics | Value | Lower 95% confidence threshold | Upper 95% confidence threshold |
| Correct classification | 0.496 | 0.436 | 0.556 |
| Incorrect classification | 0.504 | 0.444 | 0.564 |
| **Sensibility** | **0.577** | **0.481** | **0.667** |
| **Specificity** | **0.444** | **0.370** | **0.521** |
| Fraction of false positive | 0.556 | 0.480 | 0.631 |
| Fraction of false negative | 0.423 | 0.330 | 0.516 |
| Prevalence | 0.391 | 0.332 | 0.450 |
| **Positive predictive value** | **0.400** | **0.322** | **0.478** |
| **Negative predictive value** | **0.621** | **0.532** | **0.709** |
| **Positive likelihood ratio** | **1.038** | **0.838** | **1.287** |
| **Negative likelihood ratio** | **0.952** | **0.717** | **1.263** |
| Relative risk ratio | 1.055 | 0.780 | 1.427 |
| Odds ratio | 1.091 | 0.665 | 1.790 |

F .Contingency table for QIgG>QLim + 87.5

|  | CNS-AD | Other |  |
| --- | --- | --- | --- |
| QIgG>QLim + 87.5 | 16 | 20 |  |
| QIgG>QLim + 87.5 | 88 | 142 |  |
|  |  |  |  |
|  |  |  |  |
| Statistics | Value | Lower 95% confidence threshold | Upper 95% confidence threshold |
| Correct classification | 0.594 | 0.535 | 0.653 |
| Incorrect classification | 0.406 | 0.347 | 0.465 |
| **Sensibility** | **0.154** | **0.096** | **0.237** |
| **Specificity** | **0.877** | **0.816** | **0.919** |
| Fraction of false positive | 0.123 | 0.073 | 0.173 |
| Fraction of false negative | 0.846 | 0.778 | 0.914 |
| Prevalence | 0.391 | 0.332 | 0.450 |
| **Positive predictive value** | **0.444** | **0.282** | **0.607** |
| **Negative predictive value** | **0.617** | **0.555** | **0.680** |
| **Positive likelihood ratio** | **1.246** | **0.677** | **2.292** |
| **Negative likelihood ratio** | **0.965** | **0.873** | **1.067** |
| Relative risk ratio | 1.162 | 0.784 | 1.721 |
| Odds ratio | 1.291 | 0.641 | 2.601 |

Abbreviations: Q. quotient; IgG. Immunoglobulin G; **CNS-AD, Central nervous system autoimmune disease;**

QIgG, Quotient IgG. QLim, QIgG upper Limit

| Characteristics | Full population  n=266 | CNS-AD  n (%) =104 (39) | Other diagnosis  n (%) =162 (61) | OR [95%CI] | p-value |
| --- | --- | --- | --- | --- | --- |
| CSF Index scores (Table 4) |  |  |  |  |  |
| QIgG>0.0035 | 163 (61) | 64 (61) | 99 (61) | 1 [0.6-1.6] | 0.944 |
| IgG index>0.7 | 64 (24) | 37 (35) | 27 (16) | **2.8 [1.5-4.9]** | **<0.001** |
| Reiber’s formula>0 * | 150 (56) | 60 (58) | 90 (55) | 1 [0.5-1.5] | 0.732 |
| New index’s cut-off |  |  |  |  |  |
| QIgG>0.002 | 108 (40) | 49 (52) | 59 (34) | 1.4 [0.6-2.9] | 0.388 |
| IgG index>0.62 | 72 (27) | 56 (60) | 16 (9) | **3.4 [2-5.7]** | **<0.001** |
| QIgG>QLim + 87.5 | 130 (49) | 56 (60) | 74 (42) | 1.3 [0.6-2.6] | 0.480 |

**Supplementary table 6. Comparison of CSF index scores regarding CNS AD with new index’s cut-off scores**

Results are expressed as n (%)

Abbreviations: CNS AD, Central nervous system autoimmune disease; CSF, Cerebro Spinal Fluid; IgG, Immunoglobulin G; QIgG, Quotient IgG. QLim, QIgG upper Limit

* Reiber’s formula>0 corresponds to QIgG>QLim

| Disease | Literature | Our population  (Table1, n=403) | References |
| --- | --- | --- | --- |
| Anti-NMDAr | 72% | 76% | Ren et al., 2025, *BMJ Neurol Open* |
| Multiple sclerosis | 87% | 90% | Dobson et al., 2013, *J Neurol Neurosurg Psychiatry* |
| ADEM | 65% | 62% | Schwarz et al., 2001, *Neurology* |
| Neuromyelitis optica | 10% | 45% | Cacciaguerra et al., 2023, *Neurology* |
| MOG antibody-associated encephalomyelitis | 3-5% | 25% | Dubey et al., 2019, *JAMA Neurology* |
| Systemic AD with CNS involvement | 12% | 50% | Ren et al., 2025, *BMJ Neurol Open* |
| Stroke | 5% | 0 | Prüss et al., 2012, *Arch Neurol* |

**Supplementary Table 7. Comparison of ISI prevalence in literature vs. present ICU Cohort**

ICU, Intensive Care Unit; ; CNS, Central Nervous System; AD, autoimmune disease; ISI, Intrathecal Synthesis of Immunoglobulins; ADEM, Acute disseminated encephalomyelitis; MOG, Myelin oligodendrocyte glycoprotein; NMDAr, N-methyl-D-aspartate receptor.
